# Efficacy & safety of *Carica papaya* leaf extract (CPLE) in severe thrombocytopenia (≤30,000/μl) in adult dengue – Results of a pilot study

**DOI:** 10.1371/journal.pone.0228699

**Published:** 2020-02-19

**Authors:** Dipu T. Sathyapalan, Athira Padmanabhan, Merlin Moni, Binny P-Prabhu, Preetha Prasanna, Sabarish Balachandran, Sreekrishnan P. Trikkur, Soumya Jose, Fabia Edathadathil, Jagan O. Anilkumar, Rekha Jayaprasad, Gireeshkumar Koramparambil, Ravindra C. Kamath, Veena Menon, Vidya Menon

**Affiliations:** 1 Department of General Medicine, Amrita Institute of Medical Sciences, Kochi, Kerala, India; 2 Clinical Virology Laboratory, Amrita Institute of Medical Sciences, Kochi, Kerala, India; 3 Department of Medical Administration, Amrita Institute of Medical Sciences, Kochi, Kerala, India; 4 Department of Emergency Medicine, Amrita Institute of Medical Sciences, Kochi, Kerala, India; 5 Department of Allied Health Sciences, Amrita Institute of Medical Sciences, Kochi, Kerala, India; 6 Department of Integrated & Holistic Medicine, Amrita Institute of Medical Sciences, Kochi, Kerala, India; International AIDS Vaccine Initiative, UNITED STATES

## Abstract

Severe thrombocytopenia in dengue often prompts platelet transfusion primarily to reduce bleeding risk. In India, about 11–43% of dengue patients report receiving platelet transfusions which is considered scarce and expensive especially in resource limited settings. Herein, we evaluated the efficacy and safety of *Carica papaya* leaf extract (CPLE) in the management of severe thrombocytopenia (≤30,000/μL) in dengue infection. 51 laboratory confirmed adult dengue patients with platelet counts ≤30,000/μL were randomly assigned to either treatment (n = 26) or placebo (n = 24) group. By day 3, CPLE treated patients reported significantly (*p = 0*.*007*) increased platelet counts (482%± 284) compared to placebo (331%±370) group. In the treatment group, fewer patients received platelet transfusions (1/26 v/s 2/24) and their median time for platelets to recover to ≥ 50,000/μL was 2 days (IQR 2–3) compared to 3 days (IQR 2–4) in placebo. Overall, CPLE was safe and well tolerated with no significant decrease in mean hospitalization days. Plasma cytokine profiling revealed that by day 3, mean percent increase in TNFα and IFNγ levels in treatment group was less compared to that observed in placebos; (TNFα: 58.6% v/s 127.5%; *p = 0*.*25* and IFNγ: 1.93% v/s 62.6% for; *p = 0*.*12*). While a mean percent increase in IL-6 levels occurred in placebos (15.92%±29.93%) by day 3, a decrease was noted in CPLE group (12.95%±21.75%; *p = 0*.*0232*). Inversely, CPLE treated patients reported a mean percent increase compared to placebo by day 3 (143% ±115.7% v/s 12.03%± 48.4%; *p = 0*.*006*). Further, by day 3, a faster clearance kinetics of viral NS1 antigenemia occurred–mean NS1 titers in treatment group decreased to 97.3% compared to 88% in placebos (*p = 0*.*023*). This study demonstrates safety and efficacy of CPLE in increasing platelet counts in severe thrombocytopenia in dengue infections. A possible immunomodulatory and antiviral activity may be attributed to CPLE treatment. These findings merit validation in larger prospective studies.

Trial registration

Name of the registry: Clinical Trials Registry—India (CTRI)

Registration No.: CTRI-REF/2017/02/013314

## Introduction

Dengue is a mosquito borne flaviviral infection endemic to the tropics and sub-tropics. With a case fatality rate (CFR) of 2.5% and 1,300 disability-adjusted life years (DALYs) per million, dengue infections cause significant economic burden to public health systems in the endemic countries [1, 2]. According to the National Vector Borne Disease Control Program (NVBDCP), the number of dengue cases reported in India in 2017 was 153656 with 226 deaths [3]. Clinically, dengue infections usually present as mild fever that resolves rapidly. However, in some instances, it can progress to severe disease characterized by thrombocytopenia, excessive bleeding and plasma leakage, which eventually leads to shock and death. There are four distinct serotypes of dengue virus that can cause infection viz. DEN-1, DEN-2, DEN-3 and DEN-4.

The course of dengue illness is usually characterized by three phases–febrile, critical and recovery. Thrombocytopenia with coagulation abnormalities is the typical feature of severe dengue [4, 5]. Bone marrow suppression, dysfunctional megakaryocyte maturation and rapid peripheral platelet loss are some of mechanisms attributed to reduced platelet production and consequently thrombocytopenia [6]. The platelet loss in dengue is usually transient that recovers spontaneously. Although, the precise mechanistic basis of thrombocytopenia and bleeding in dengue infections are still unknown, one of the mechanisms proposed involves inhibition of bone marrow progenitor cell proliferation and its functions [7]. Another possibility is the bone marrow hypoplasia and attenuation of megakaryocyte development during the acute phase of DENV infection, causing suppression of platelet production [8]. DENV infection can also lead to apoptosis of bone marrow progenitor cells, destruction of stromal cells or dysregulation of thrombopoiesis [9]. Haemorrhagic manifestations in dengue are usually the result of a complex interplay of peripheral platelet dysfunction and loss and/or disseminated intravascular coagulation. Plasma leakage and coagulation irregularities characterize severe dengue [10, 11]. The genetic background of the patient, immune response to the infection and characteristics of the infecting virus, all contribute to severe dengue. Studies have shown that the non-structural protein (NS1) of DENV when expressed in infected cells can upregulate the membrane bound Tissue Factor (TF) that activates platelets [10, 12]. Initiation and activation of coagulation and inflammatory pathways lead to the release of pro-inflammatory cytokines like TNF-α, IFN-γ and IL-6. Elevated levels of TNF-α has been shown to be associated with hemorrhagic symptoms. Thrombocytopenia can also result from endothelial sequestration, wherein platelets increasingly attach to von-Willebrand factor (vWF) on vascular endothelial cells, resulting in small clogs in microcirculation and low levels of platelets in peripheral blood [12, 13].

Lack of an effective antiviral or vaccine against dengue infections necessitates its management to be primarily supportive and symptomatic. Prophylactic or therapeutic platelet transfusions are sometimes employed to manage dengue induced thrombocytopenia with or without serious hemorrhagic manifestations; however, its efficacy in such situations is controversial [14]. There are numerous reports of natural remedies for management of thrombocytopenia in dengue. In many countries, oral administration of a hot-water extract of *Carica papaya* leaves acts as an antipyretic and as a natural remedy to treat dengue fever with thrombocytopenia [15]. This extract, in some experimental studies has demonstrated erythrocyte stabilization potential that prevents hemolysis [16,17]. It is shown to also exert a direct action on platelets by preventing its aggregation especially during dengue infections [18]. A study by *Sanath Hettige et al* performed in Sri Lanka confirmed a pronounced increase in platelet and leukocyte counts with CPLE treatment [19]. Similarly, *Soobitha Subenthiran et al* have shown that *Carica papaya* leaves juice (CPLJ) appreciably hastens the rate of increase in platelets in dengue fever and DHF [20]. Further, they observed a significant increase in the expression levels of ALOX 12 and PTAFR genes—two genes known to play a major role in hematopoiesis. More recently, in-vitro studies by *Sharma et al* have demonstrated a direct antiviral action of CPLE in DENV infected THP-1 cells wherein a decrease in the intracellular viral load and NS1 protein expression was noted with CPLE treatment [21].

Although evidence for a beneficial role of platelet transfusions in the management of severe thrombocytopenia is currently lacking, studies have shown that about half of treating physicians, in India, fearing the risk of bleeding, continue to use platelet transfusions prophylactically to manage severe thrombocytopenia [5]. However, given the high cost and scarcity of blood products and potentially risky nature of platelet transfusions, their inappropriate use can easily escalate the already increasing healthcare cost of dengue. The safety and efficacy of Caripill (CPLE) to increase platelet counts in mild to moderate thrombocytopenia has been established. However, the role of CPLE in the management of severe thrombocytopenia (<30,000/μl) in dengue, has not yet been evaluated. In this study, we investigated the safety and efficacy of CPLE in improving platelet counts and reducing the need for platelet transfusions in the management of dengue patients with severe thrombocytopenia.

## Materials and methods

### The study protocol and supporting CONSORT checklist are available as supporting information; see S1 Checklist and S1 Protocol

#### Study design & patients

This double-blind, placebo-controlled, randomized, prospective study was conducted at a tertiary care hospital in India. Adults patients (≥ 18yrs of age), presenting with fever, a peripheral platelet count of ≤ 30,000/μL and positivity on a dengue point-of-care diagnostic test (SD BIOLINE Dengue DUO^®^, Standard Diagnostic Inc., Korea) were eligible for study enrollment, after duly signing an informed consent form. The study exclusion criteria included children (<18 years), pregnancy, lactation, patients on steroids or other indigenous medications, patients who received platelet transfusion recently, and patients who had alanine aminotransferase (ALT) levels >150 U/L or creatine kinase (CK) >1000 U/L and myopathy.

Ethics approval for this study was obtained from the Institutional Review Board (IRB) of the hospital on 30^th^ August 2016 and trial was registered with the Clinical Trial Registry of India (CTRI-REF/2017/02/013314) on 31^st^ August 2017. Although the trial was submitted to CTRI immediately after IRB approval, procedural issues resulted in registration to be finalized only on 31^st^ August 2017 which was before data analysis. All study procedures and conduct were in accordance to good clinical practice and the Declaration of Helsinki.

#### Subject recruitment and follow-up

The study was conducted from 1^st^ November 2016 to November 1^st^ 2017. The first patient was enrolled on 5^th^ Dec 2016. Study patient recruitment continued till 17^th^ Oct 2017. Follow-up evaluation for liver dysfunction was done by assessing the levels of liver enzymes (AST, ALT) two weeks post the last treatment dose. The study was completed on 31^st^ October 2017.

#### Randomization and masking

Enrolled patients were randomly assigned in (1:1) ratio to receive either CPLE (Caripill) or placebo tablets three times daily for 5 days. Online randomization software was used for patient assignment and random permuted block of length four were used to ensure balance over time. The treatment allocations were blinded to the clinical investigators until the completion of the study except the randomization statistician. Packages of CPLE (Caripill) and placebo were coded at the manufacturing site and sent to randomization statistician. The placebo contained microcrystalline cellulose, sodium starch glycolate, caramel color, croscarmellose sodium, stearic acid, colloidal silicone dioxide, crospovidone, magnesium stearate, polyethylene glycol, hydroxypropyl methyl cellulose, talc and titanium dioxide. It was administered as 1 tablet (1100mgs) three times a day orally for 5 days. Concealment of allocation was centralized where-in the recruiting physician provided informed consent and other details of the enrolled study subject to a study coordinator who was not involved with patient care nor was present at recruitment site. After ensuring completeness and correctness of the provided information, the study coordinator sent out sealed study medication packages to the recruiting physician. Identification of study medication package was concealed from the physician by masking the medication label. All study participants received no other medication other than the study drug and placebo Rest of the study team, including the patients, was blinded until completion of the study. A structured case report form (CRF) was used to obtain all patient details which were then entered into an Excel database. Investigators had no access to the database until study completion.

#### Study assessments

*Clinical evaluations*. All study patients were administered study medications immediately (within 30 mins) after a baseline blood sample for study investigations were collected. While treatment group received CPLE tabs 1100 mg three times a day for 5 days, the control group received visually matched placebo with same dosing schedule. Study patients were followed by the treating physician, daily until discharge, and all signs and symptoms were recorded in CRF. Clinical care decisions such as parenteral fluid and blood product treatment was at the discretion of treating team. The study team did not in any way influence the treatment particulars in the enrolled patients. Biochemical and hematological laboratory assessments were performed daily during the study period.

*Laboratory evaluations*. Venous blood samples were collected at day 0 (prior to study drug administration), day 3 and day 5 after starting treatment. Plasma samples were collected and stored frozen in multiple aliquots at −80°C until analysis. Dengue NS1, IgM and IgG in plasma was determined using the Dengue Early ELISA, Dengue IgM capture ELISA and Dengue IgG Capture ELISA kit (Panbio, Brisbane, Queensland, Australia) according to the manufacturer's instructions. Plasma cytokine levels of Tumor Necrosis Factor α (TNF-α), Interferon γ (IFN-γ), IL-4 and IL-6 were measured using Single-Analyte ELISArray Kits (Qiagen, Inc., Valencia, CA). Viral RNA was extracted from clinical samples using QIAamp viral RNA kit (Qiagen, Inc., Valencia, CA). RT-PCR for detecting and typing of dengue viruses was carried out according to *Robert S Lanciotti et al*. (1992) [22].

*Safety assessments*. Adverse events, if any, during the study period were appropriately recorded and documented in the safety assessment form. Safety assessment included monitoring of vital signs, laboratory evaluations–CBC–LFTs–Renal Function Tests during the study period with a follow-up at 2 weeks after stopping the medications. A satisfactory clinical status as determined by the attending physician entailed study discharge.

#### Study outcomes

The primary efficacy endpoints were comparison of change in platelet counts and hematocrit values during hospital stay between treatment and placebo groups. Additionally, frequency and type of blood product, more specifically platelet transfusions, required to manage thrombocytopenia during the course of illness was compared between the two study arms.

Safety and tolerability were evaluated by comparing the proportion of patients with any adverse events between the two groups. Incidence of bleeding episodes in study subjects was also assessed by clinical evaluation. Secondary study endpoints included evaluation of changes in platelet counts in study subjects based on their phase of dengue illness and infection status. Length of hospital stay of study subjects in both arms also compared. As exploratory endpoints, we examined the infection induced inflammatory responses and NS1 clearance in a sub-cohort of 20 subjects, 10 from each group. Changes in the kinetics and levels of cytokines in study subjects were compared.

#### Sample size determination and analysis

Our initial sample size calculation was performed using the online sample size calculator provided at https://clincalc.com/stats/samplesize.aspx. Our analysis was based on an earlier study by *Ajeeth kumar Gadhwal et al* (http://www.japi.org/june_2016/03_oa_effect_of_carica.pdf) where in the minimum sample size calculated for the randomized controlled trial comes to 32 (16 in each arm) at 0.05 alpha level and 80% power. A target sample size of 100 patients was initially estimated taking into consideration the high incidence of dengue cases, which was reaching almost epidemic proportions, during the monsoon season in the state of Kerala in the study year. However, as the study progressed, it was observed that a substantial proportion of the dengue patients that were being admitted had already consumed some form of indigenous preparations of papaya leaf extracts and therefore were not eligible for study enrolment. In addition, the fraction of dengue patients with severe thrombocytopenia (platelet count of <30,000/μL)–an important study inclusion criterion, being admitted to our hospital were fewer than expected which adversely affected the study recruitment rate. As the monsoons abated, the dengue positive cases being admitted to the hospital dwindled and consequently study recruitment process further slowed down. As the study was time bound, with due consultations with the sponsors and other study team members, we stopped recruitment when we reached a sample size of almost 50 patients which was more than the minimum sample size determined at the start of the study.

#### Statistical analysis

Data analyses were performed using Graphpad Prism 8.0 (San Diego, California, USA) statistical software. Demographic, clinical and laboratory variables were compared either using X^2^-test for categorical variables and Student’s t-test for continuous variables. The primary outcome data was averaged and compared for both groups using the Student’s t- and Mann-Whitney U-tests. For all variables, significance was assigned using a two-sided p-value ≤0.05. A Kaplan-Meier curve was used to measure the time to event, in this case ‘time to platelet count > 50 000/μL’ and log-rank test was performed for group comparisons. The Cox proportional hazards model to estimate hazard ratio (HR) with 95% CI for ‘time to platelet count > 50 000/μL’ and ‘length of hospital stay’ was also done.

## Results

### Study cohort

**Fig 1:**
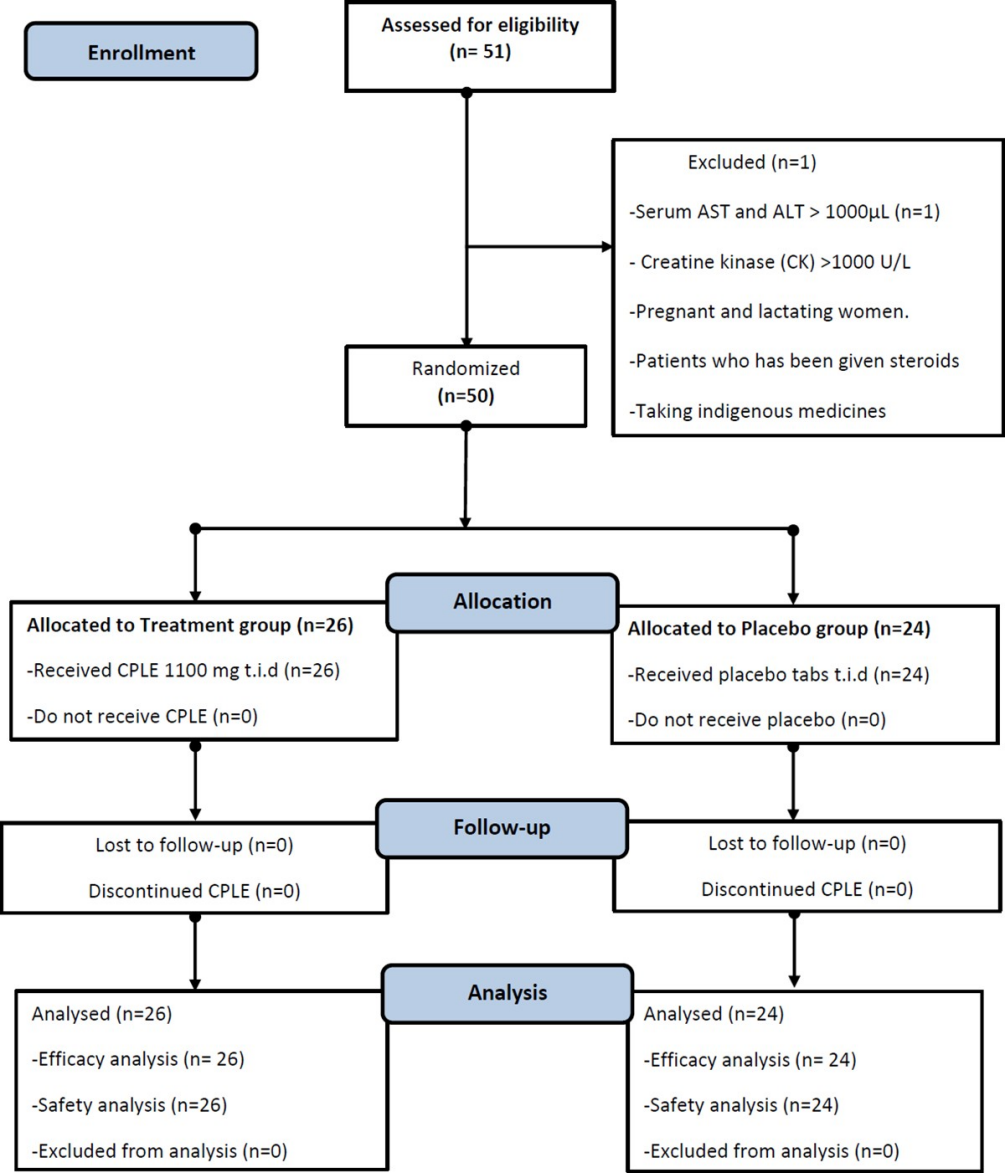
Study subject disposition.

Of the 51 patients screened and enrolled, one patient with elevated AST (>1000 IU/ml) was excluded. Rest were randomly assigned to either treatment group (n = 26) or placebo (n = 24). The disposition of study subjects is depicted in **Fig 1**.

The baseline demographic, clinical and laboratory characteristics of study cohort were similar in both groups **Tables 1 & 2**. Majority of the study patients were in critical phase of dengue illness; 62% (16/26) in CPLE group and 50% (12/24) in the placebo group. Secondary dengue was commonly observed; 69% (18/26) in CPLE and 71% (17/24) in the placebo group. In cases where viral RNA was detected, the predominant serotype was DENV-2 (60%; 14/23). Fever and lethargy were commonly observed in both groups, except for a higher incidence of abdominal pain in treatment group [19%; 5/26 v/s 8%; 2/24) and persistent vomiting in placebos [21%; (5/24) v/s 19%; (5/26)]. The clinical management of study patients was similar in both groups with only few patients receiving blood product transfusions. Crystalloids were used for fluid management and no fatality was recorded in both groups; **Tables 1 & 2**.

**Table 1:** Demographic and baseline clinical features of study subjects.

Subject Variables	Treatment group (n = 26)	Placebo group (n = 24)
**Demographics**		
Age (yrs)	51(16)	54 (14)
Gender		
Female	7 (27%)	12 (50%)
Male	19 (73%)	12 (50%)
**Clinical Features**		
Fever (in days)	6 (5)	5 (2)
Abdominal pain	5 (19%)	2 (8%)
Persistent vomiting	5 (19%)	5 (21%)
Lethargy	25 (96%)	24 (100%)
Mucosal bleed	2 (7%)	3 (12.5%)
Restlessness	1 (4%)	0
Rapid breathing	1 (4%)	0
Liver enlargement	1 (4%)	1 (4%)
**Infection details**		
**Phase of illness**		
Febrile	3 (11.5%)	2 (8%)
Critical	16 (61.5%)	12 (50%)
Recovery	7 (27%)	10 (42%)
**Infection Status**		
Primary	8 (31%)	7 (29%)
Secondary	18 (69%)	17 (71%)
**Serotype**		
1	4 (15%)	3 (12.5%)
2	7 (27%)	7 (29%)
3	0	1 (4%)
4	0	0
Co-infection	2 (8%)	1 (4%)
Negatives	13 (50%)	12 (47%)
**Viral Antigen**		
NS1Positives	16 (61.5%)	10 (42%)
IgM Positives	25 (96%)	24 (100%)
IgG Positives	24 (93%)	21 (87.5%)
**Outcome**		
Discharged	26 (100%)	24 (100%)
Death	0	0
Length of stay (days)	5 (1)	5 (1)
**Treatment details**		
**Platelet products**		
Within 48 hrs	12 (46%)	5 (21%)
After 48 hrs	1 (4%)	2 (8%)
Crystalloids	21 (81%)	13 (54%)
Colloids	0	0

Data are n (%) and mean (± s.d)

**Table 2:** Baseline haematological and biochemical values of study subject.

Laboratory parameters	Treatment group (n = 26)	Placebo group (n = 24)
**Haematology**		
WBC (K/μL)	5 (2)	7 (5)
Neutrophils (%)	45 (17)	48 (15)
Monocytes (%)	6 (5)	9 (12)
Lymphocytes (%)	47 (15)	42 (14)
Eosinophil (%)	1 (2)	1 (1)
Basophils (%)	1 (0.6)	1 (0.3)
Platelets (K/μL)	20 (5)	22 (8)
RBC (M/μL)	5 (1)	5 (1)
Haemoglobin (g/dL)	14 (2)	14 (3)
HCT (%)	43 (5)	41 (6)
MPV (%)	11 (1.4)	11 (1.2)
RDW (%)	13 (1)	13 (1)
**Biochemistry**		
Serum Sodium (mmol/L)	134 (4)	135 (4)
Serum Potassium (mmol/L)	4 (1)	4 (1)
Blood Urea (mg/dL)	22 (8)	22 (8)
Serum Creatinine (mg/dL)	1 (0)	1 (0)
Serum Bilirubin (mg/dL)	1 (0)	1 (0)
SGPT (IU/L)	141 (162)	124 (105)
SGOT (IU/L)	228 (259)	199 (207)
ALP (IU/L)	70 (31)	99 (75)
Serum Albumin (g/dL)	4 (0)	4 (1)
Serum Globulin (g/dL)	3 (0)	3 (1)
Serum Calcium (mg/dL)	7 (2)	8 (0)
APTT (s)	44 (7)	43 (11)
PT (s)	14.60 (1.04)	14.10 (1.40)
INR	1.00 (0.09)	0.96 (0.12)

Data are mean (SD)

### Efficacy outcomes

#### Platelet counts

**Fig 2:**
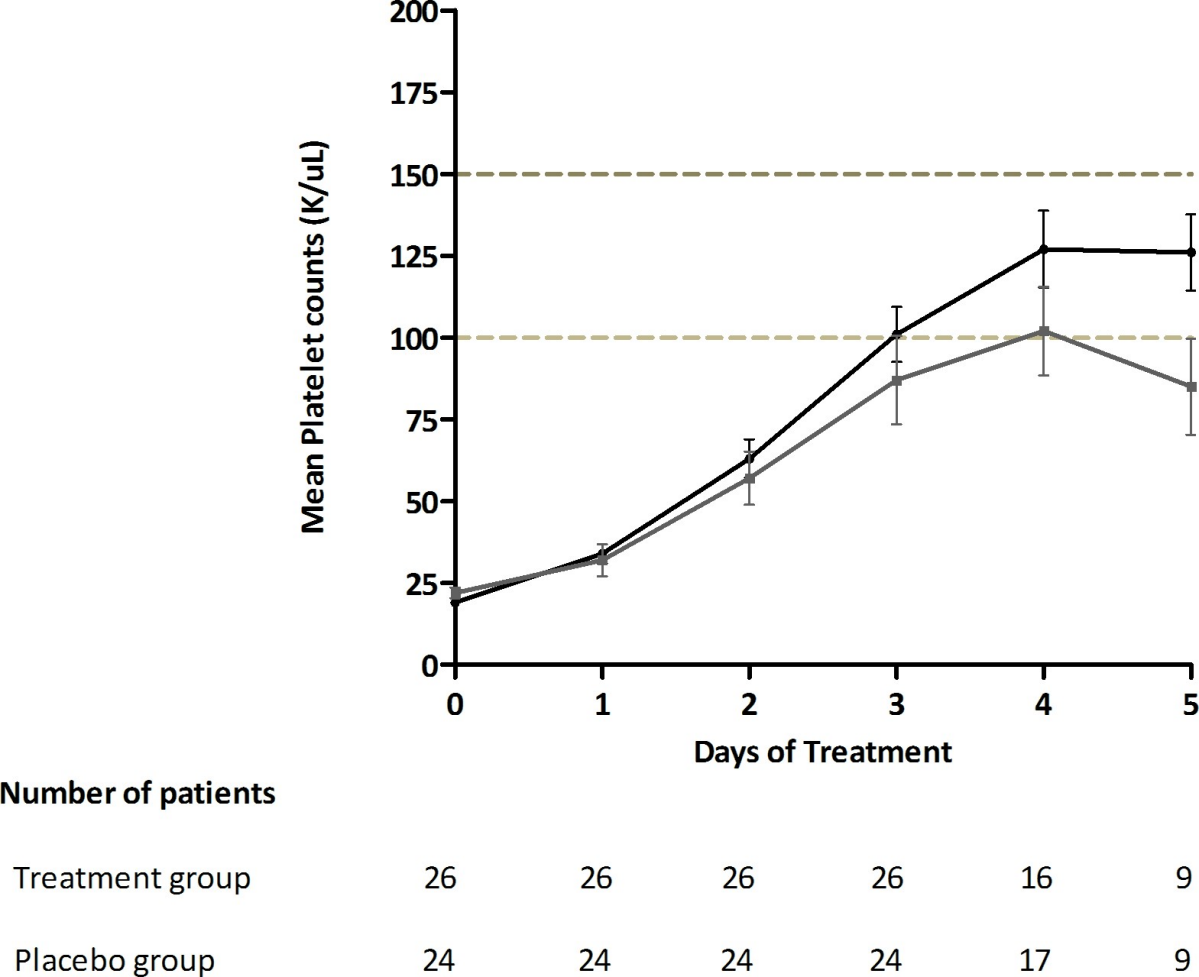
Increase in the mean (± s.e.m) platelet counts during course of treatment in the treatment group (black) and Placebo group (grey) subjects.

**Fig 3:**
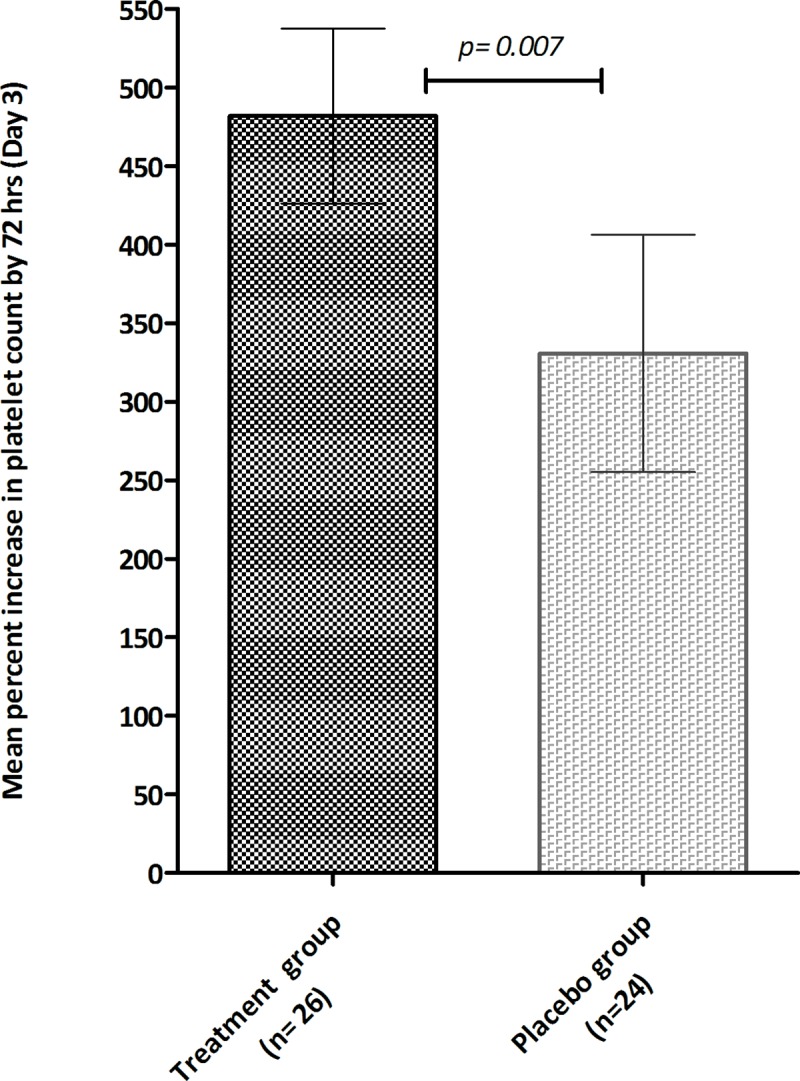
Mean (± s.e.m) percent increase in platelet count post treatment (day 3) in treatment group (black) and placebo group (grey) subjects.

**Fig 4:**
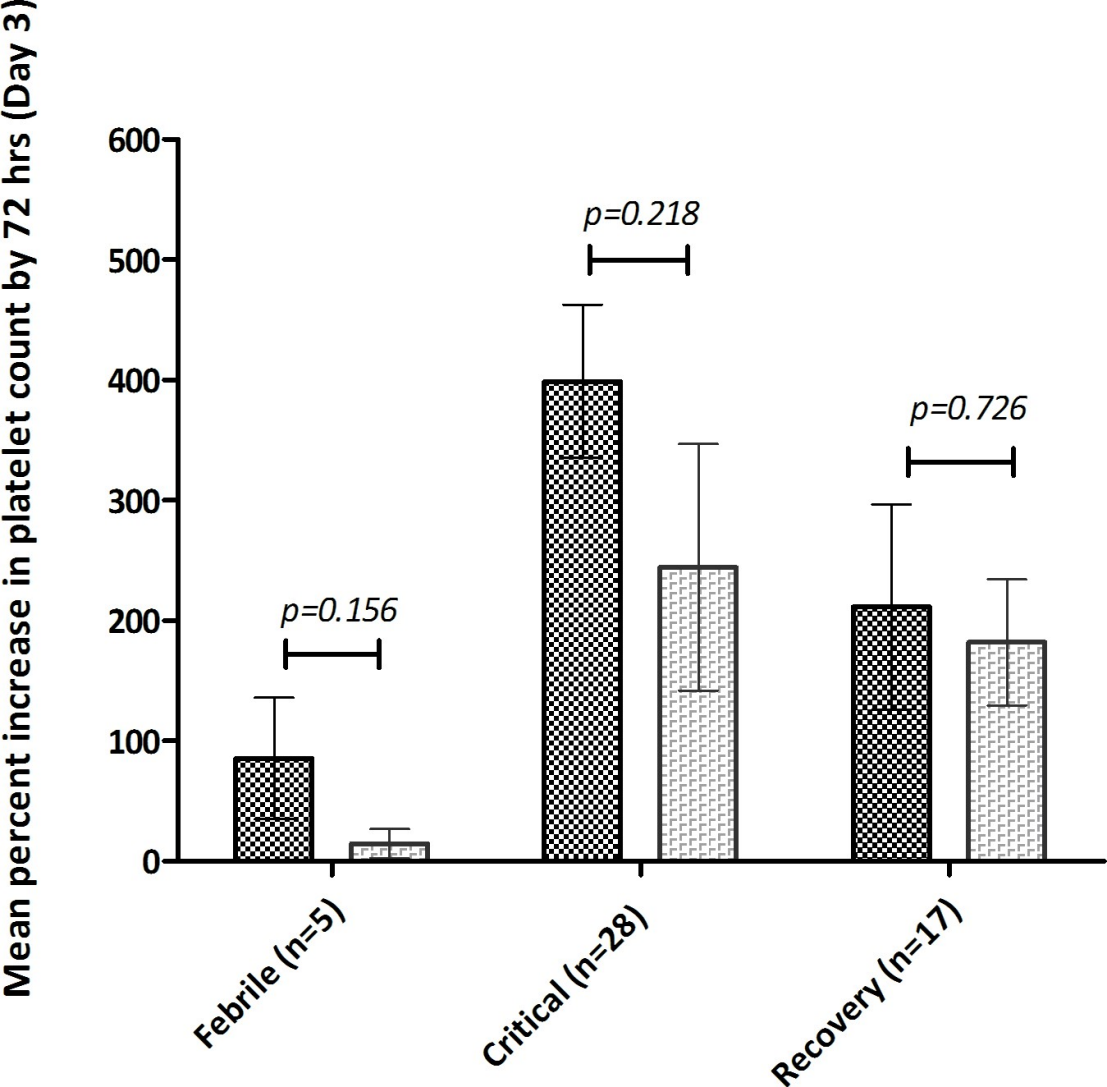
Mean (± s.e.m) percentage increase in platelet count post treatment (day 3) in treatment group (black) and placebo group (grey) subjects in the febrile, critical and recovery phases of dengue illness.

**Fig 5:**
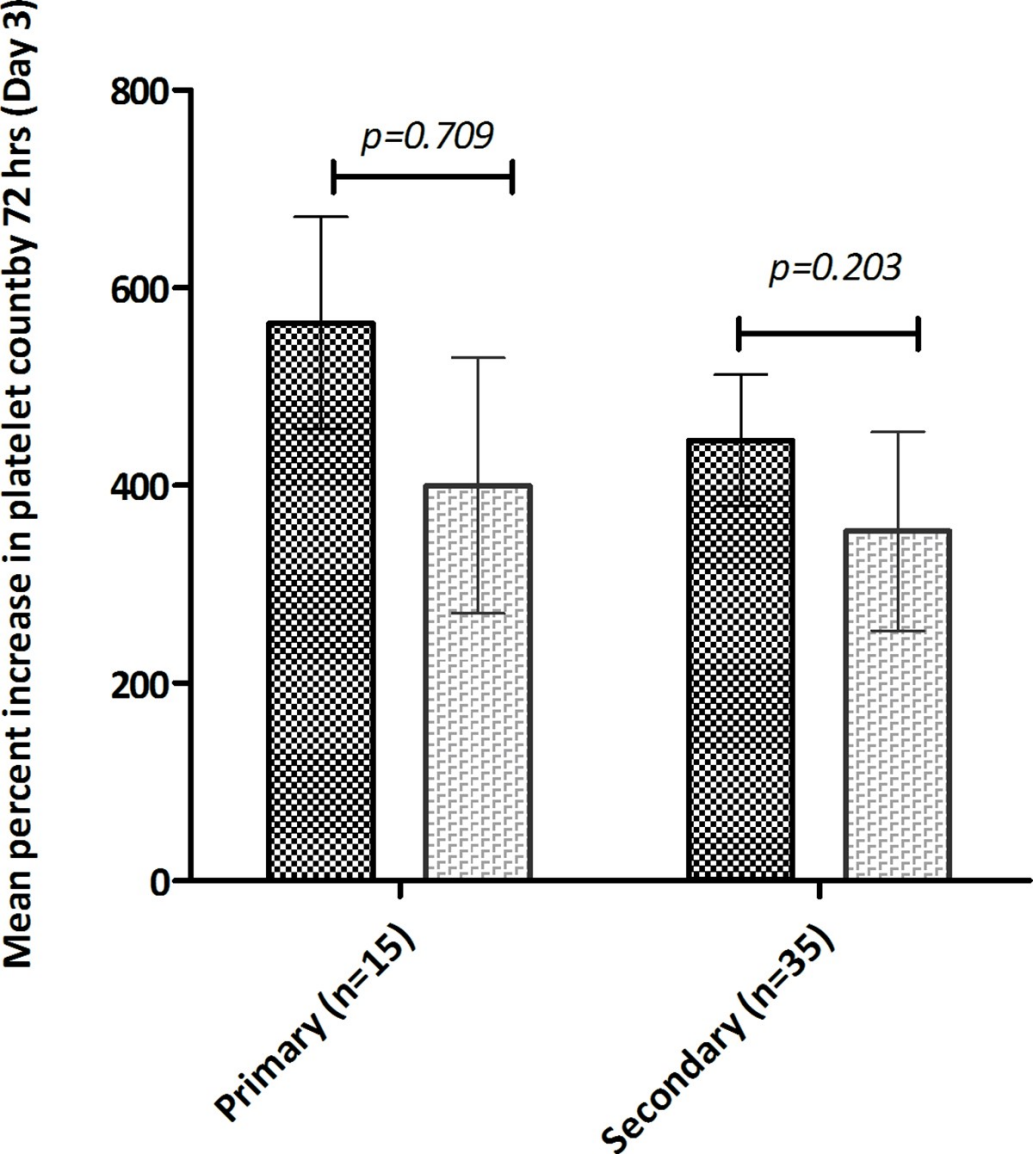
Mean (± s.e.m) percentage increase in platelet count post treatment (day 3) in treatment group (black) and placebo group (grey) subjects with primary and secondary dengue infection.

**Fig 6:**
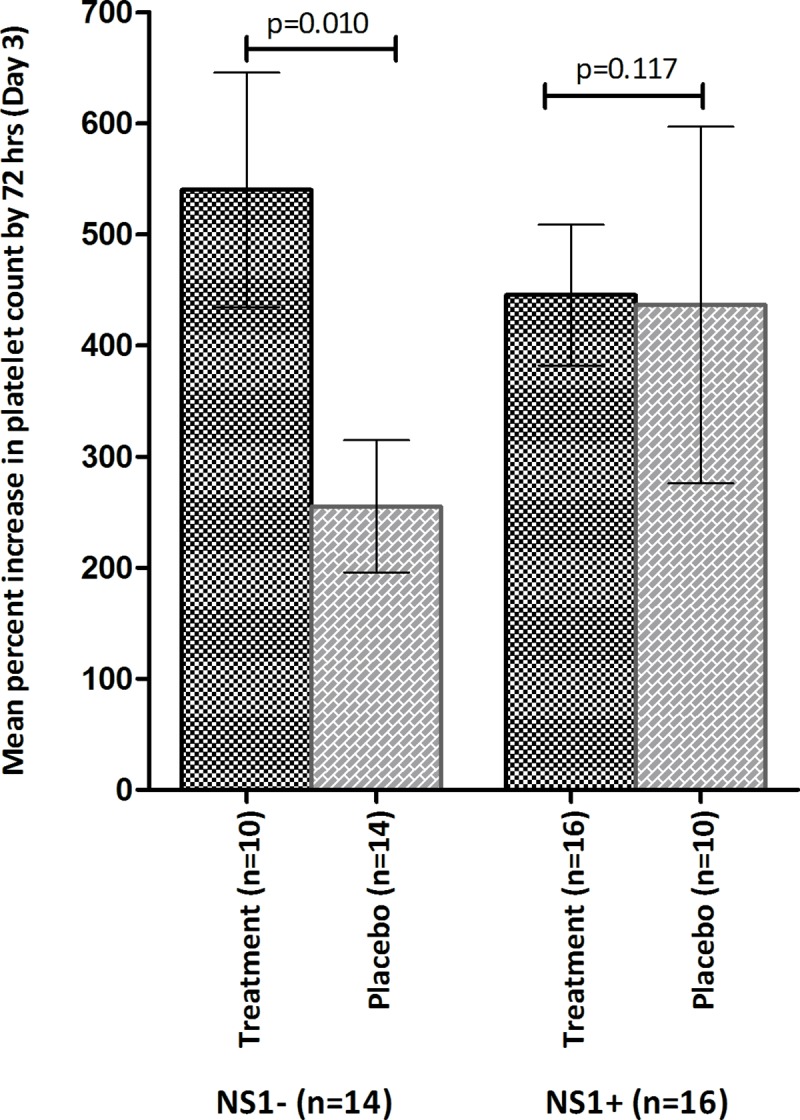
Mean (± s.e.m) percentage increase in platelet count post treatment (day 3) in treatment group (black) and placebo group (grey) in the NS1 (+) and NS (-) subgroup of study subjects.

The treatment group showed a more rapid rise in platelet counts. The changes in mean (±s.e.m) platelet counts over treatment period is depicted in **Fig 2**. Although the treatment group had a lower baseline mean (±s.e.m.) platelet count (19,000/μL±6000/μL) compared to controls (22,000/μL±8000/μL) (p = 0.37), by 72 hrs a significant percent increase in platelet numbers was noted in the CPLE group; (482%±284 v/s 331%±370; p = 0.007); **Fig 3**. The increase in platelet count in subjects stratified according to their phase of dengue illness is shown in **Fig 4**. Fewer patients (n = 5) were in their febrile phase compared to those in critical phase (n = 28) or in recovery phase (n = 17). Irrespective of the illness phase, higher mean (±s.e.m) platelets counts were observed in the treatment group, though this increase was not significant. The percent increase in platelet counts by 72 hrs was more pronounced in patients in critical phase (398.9%±63.63% v/s 244.3%±102.6%; *p = 0*.*22*). Likewise, CPLE treatment did not significantly increase the mean (±s.e.m) platelet count by 72 hrs in either primary or secondary dengue infections; 564.5%±107.3% in study subjects with primary infection (p = 0.709) and 445.8% ± 66.64% with secondary dengue (p = 0.203); **Fig 5**. However, in the NS1 negative subgroup, patients in the treatment group showed a significant increase in platelet counts (540%±105.5 v/s 255%±59.46; p = 0.0104), no such significance was observed in the NS1 positive subgroup of patients; **Fig 6**.

**Fig 7:**
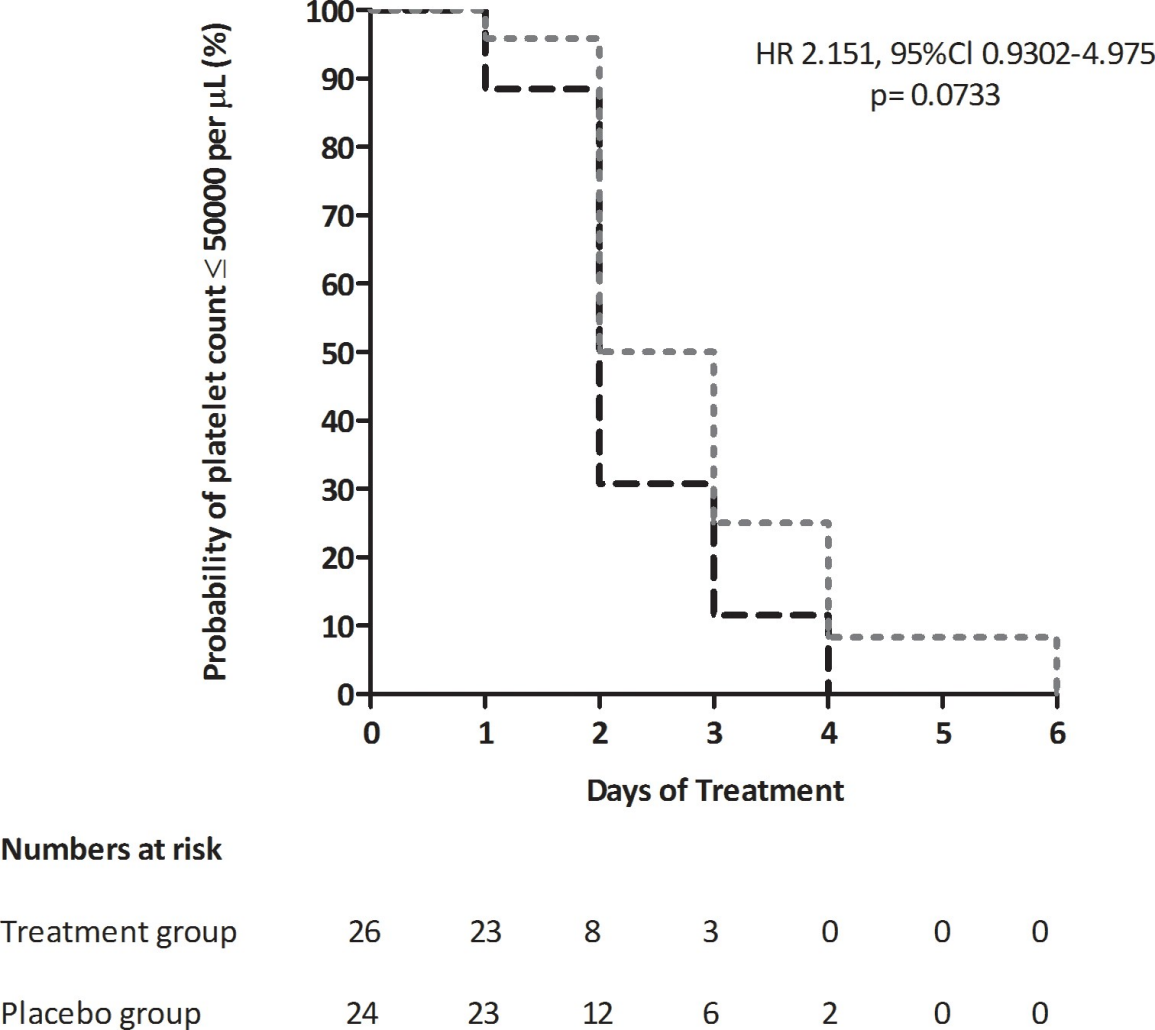
Kaplan-Meier estimates of probability of platelet count of ≤ 50000 per μL by day 6 in treatment group (black) and placebo group (grey). HR denotes hazard ratio.

Evaluation of time required for the platelet count to increase to ≥ 50,000/μL within each group is shown in **Fig 7**. The median time to attain a platelet count of ≥ 50,000/μL was less in the treatment group *i*.*e* 2 days (IQR 2–3) versus 3 days (2–4) in placebos. Also, fewer proportions of subjects from treatment group were at risk *i*.*e* still had their platelet counts <50,000/μl by day 2(HR 2.151, 95%Cl 0.9302–4.975; p = 0.0733).

#### Hematocrit values

**Fig 8:**
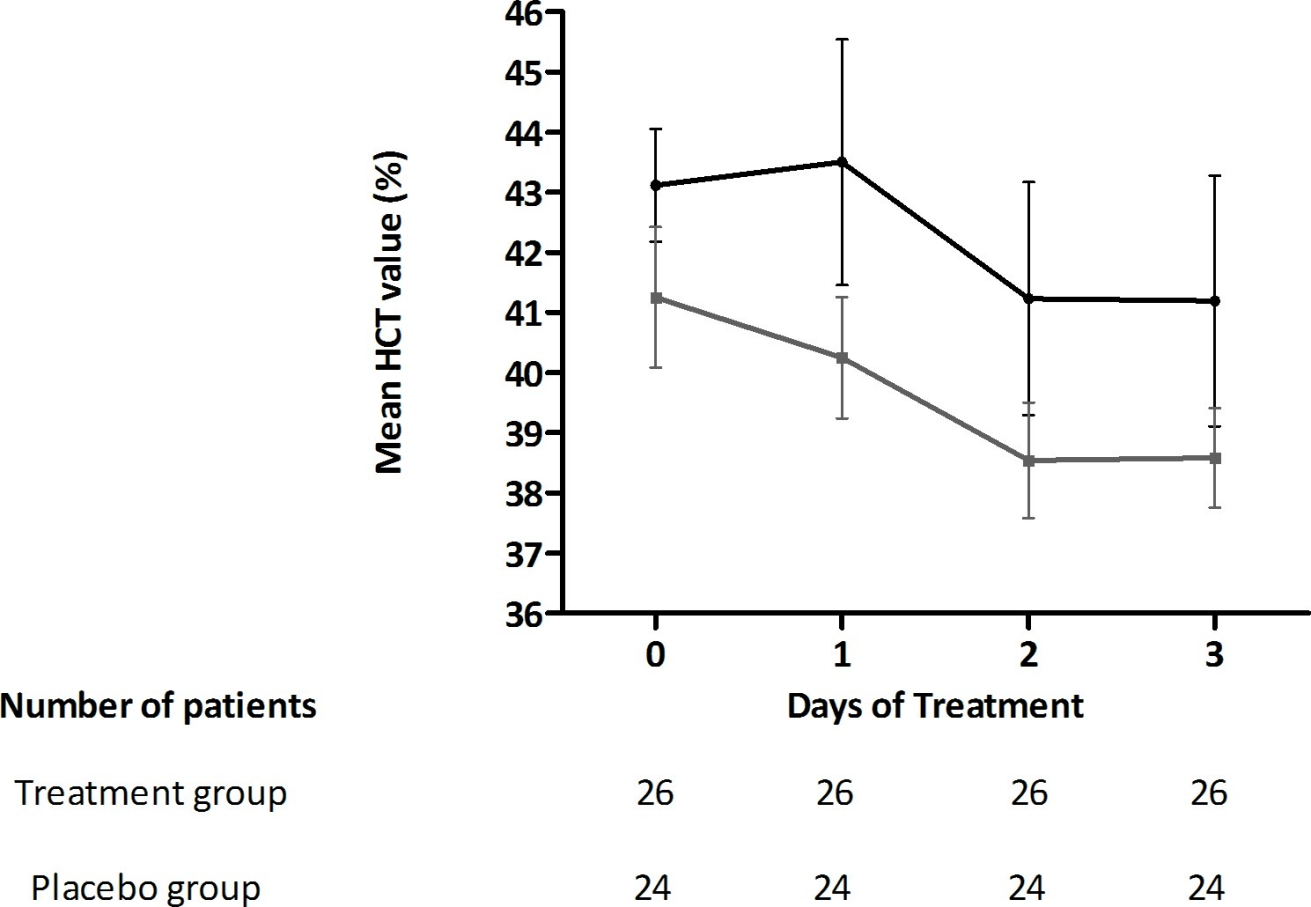
Increase in the mean (± s.e.m) hematocrit values during course of treatment in the treatment group (black) and placebo group (grey) subjects.

**Fig 9:**
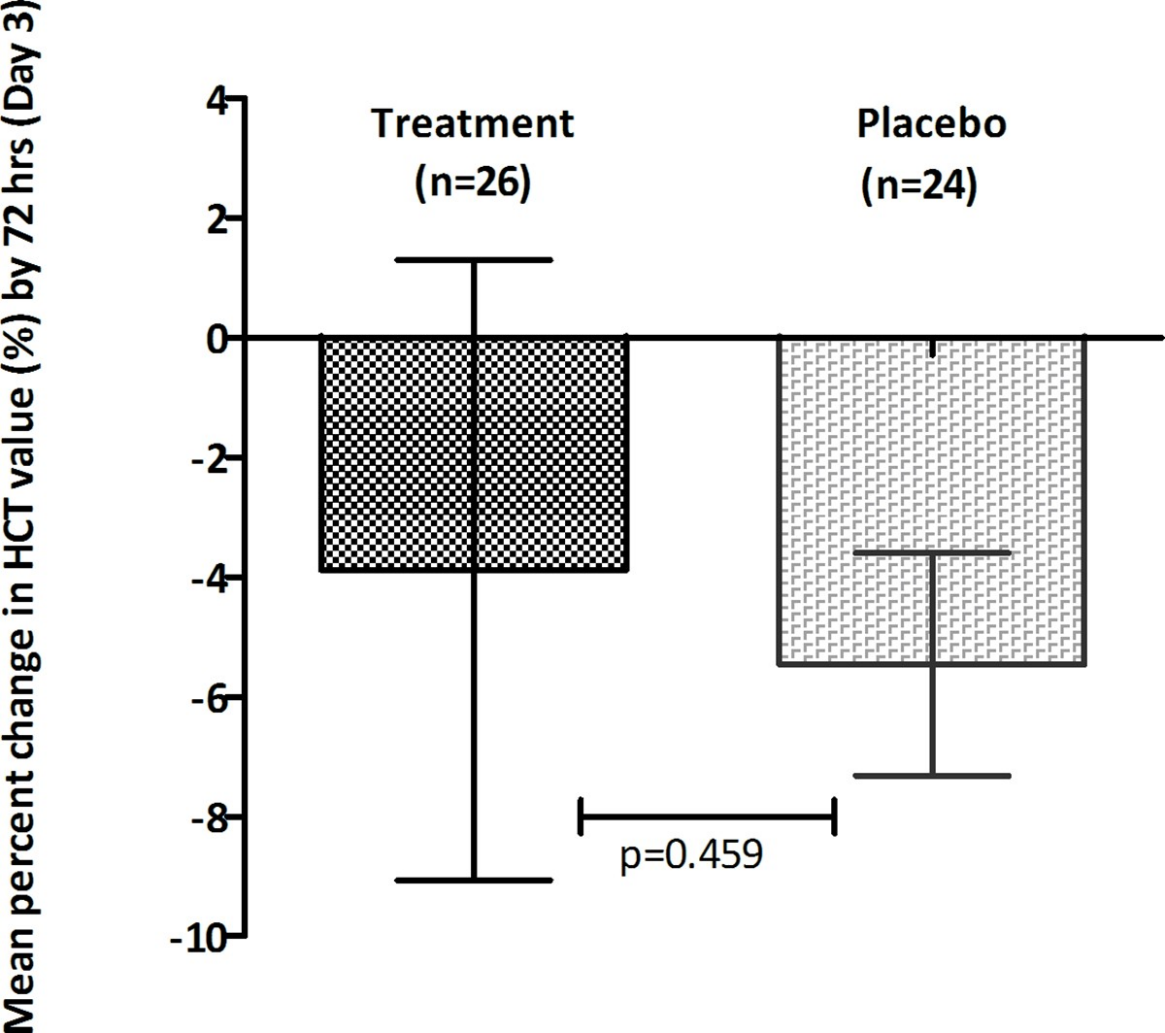
Mean (± s.e.m) percent decrease in HCT value (%) post treatment (day 3) in treatment group (black) and placebo group (grey) subjects.

No significant difference in hematocrit values was observed between the two groups indicating CPLE treatment may not significantly t influence HCT values in both groups **Figs 8 & 9**.

#### Length of hospital stay

Although no significant difference in the average (±s.e.m) length of hospitalization (5 days±1) was observed between the two groups; **Table 1**, a greater proportion of patients with severe thrombocytopenia in the treatment group (34.6%) were discharged by day 4 compared to placebo (25%); **Table 3**.

**Table 3:** Days of hospitalization of patients in treatment and placebo group.

Days of hospitalization	Treatment group (n = 26) (n%)	Placebo group (n = 24) (n%)
0	(-)	(-)
1	(-)	(-)
2	0	1 (4.2)
3	1 (3.8)	1 (4.2)
4	9 (34.6)	6 (25.0)
5	6 (23.1)	7 (29.2)
6	8 (30.8)	8 (33.3)
7	2 (7.7)	0
8	0	1(4.2)

#### Blood product usages

At baseline, only 10% (5/50) of study subjects presented with mild clinical bleeding. Usage of platelet transfusions was not significantly different in both groups; 31% (8/26) of treatment group and 17% (4/24) of controls received platelet transfusions; **Table 4**. However, by day 3, none of the patients in the treatment group required platelet transfusions, whereas 17% (1/6) of controls had persistent transfusion requirement.

**Table 4:** Platelet transfusion details of study subjects in treatment and placebo groups.

Treatment Days	Single Unit Platelet Received			
	Treatment group (n = 26)		Placebo group (n = 24)	
	Amount (SUPs)	Number of Patients	Amount (SUPs)	Number of Patients
Day 0	60	8	58	4
Day 1	42	5	18	2
Day 2	11	3	0	0
Day 3	0	0	6	1
Day 4	0	0	0	0
Day 5	0	0	0	0

### Exploratory outcomes

#### Cytokines

**Fig 10:**
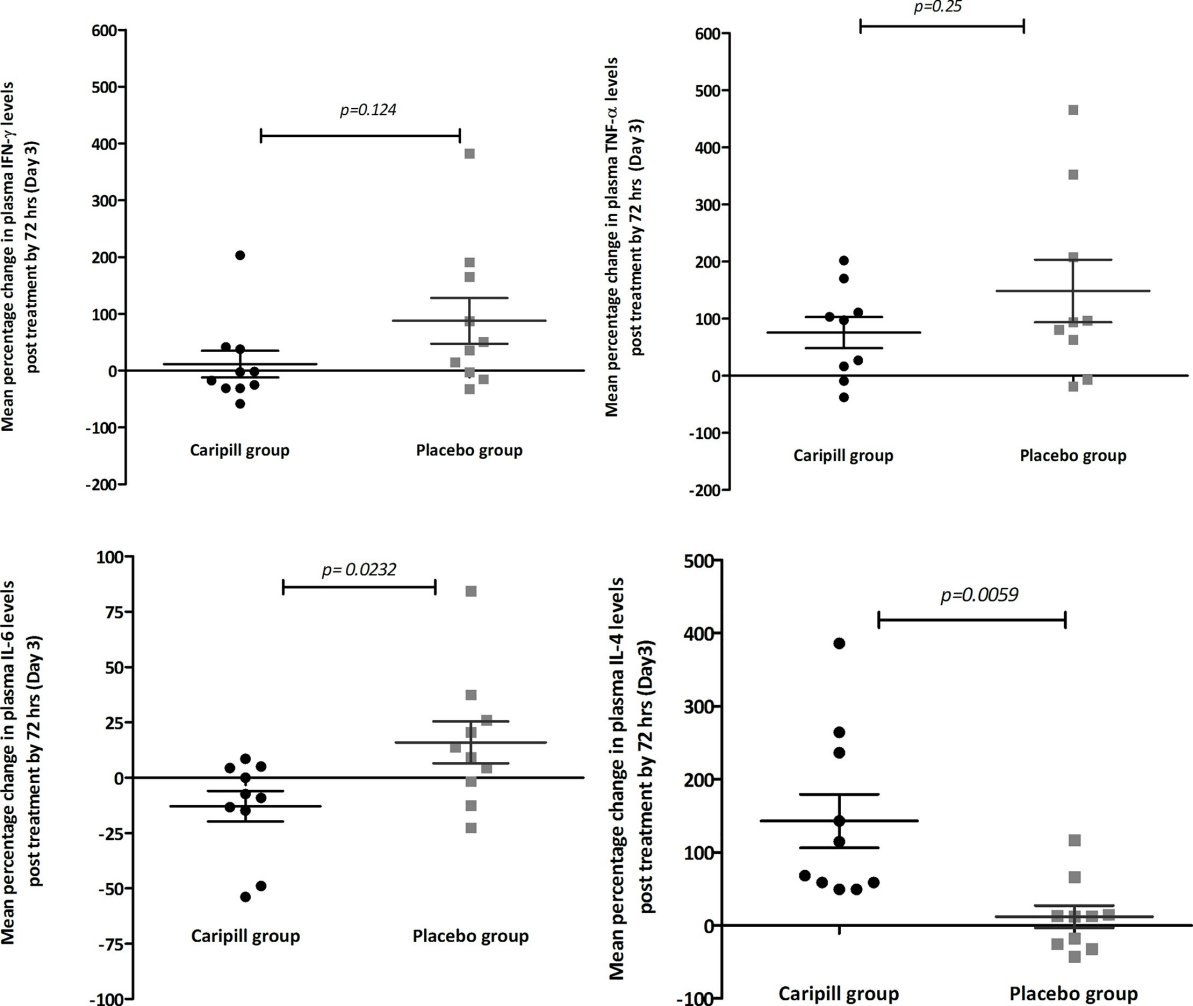
Mean percentage (±s.e.m) changes in plasma cytokine levels by 72 hrs (day 3) in the treatment group (black) and placebo group (grey) (a) IFN-γ (b) TNF-α (c) IL-6 (d) IL-4.

To assess the effect of CPLE treatment on dengue induced systemic inflammatory response, circulating plasma levels of inflammatory cytokines like TNFα, IFN-γ, IL-6, and IL-4 were estimated in a sub-cohort of 20 study subjects. The pretreatment (day 0) and 72 hrs post treatment (day 3) plasma cytokine levels were measured (**Table 5**) and the percent change in each of these cytokines by day 3 between the two groups were evaluated **Fig 10A, 10B, 10C and 10D**. As shown in **Fig 10A**, the percent increase in plasma IFNγ levels by 72 hrs was notable in the placebo group compared to treatment group, although this difference was not statistically significant. Likewise, comparative evaluation of the mean percentage difference in plasma TNFα levels by 72 hrs showed a greater but insignificant increase in placebo than in treatment group **Fig 10B**. On the other hand, CPLE treatment resulted in a mean percent decrease of 18% in contrast to a 13.07% increase in the IL6 levels in the placebo subgroup (p = 0.023);**Fig 10C**. A similar inverse trend was also observed in IL4 levels. However, unlike that observed in IL6, CPLE treatment showed a mean percent increase of 143% (±36.6) compared to 12.03% (±15.31) in the placebo patients (p = 0.0059); **Fig 10D**.

**Table 5:** Mean (±se.m) plasma levels of cytokines measured on day 0 (pre-treatment) and day 3 (post-treatment) in study subjects (n = 20).

Parameters	Treatment group (n = 10)			Placebo group (n = 10)		
	Day 0	Day 3	p value	Day 0	Day 3	p value
IFN-γ (pg/mL)	41.09 (± 18.03)	38.77 (±15.52)	0.864	35.0 (± 12.51)	57.73 (± 28.67)	0.044
TNF-α (pg/mL)	48.38 (± 26.03)	89.46 (± 40.69)	0.1	52.12 (± 41.42)	119 (±98.77)	0.089
IL-6 (pg/mL)	22.96 (± 14.09)	18.80 (± 9.93)	0.239	20.27 (± 3.12)	22.92 (± 4.07)	0.193
IL-4 (pg/mL)	2.03 (± 0.75)	2.42 (± 1.15)	0.284	1.88 (±0.48)	2.02 (± 0.75)	0.959

#### NS1 clearance

**Fig 11:**
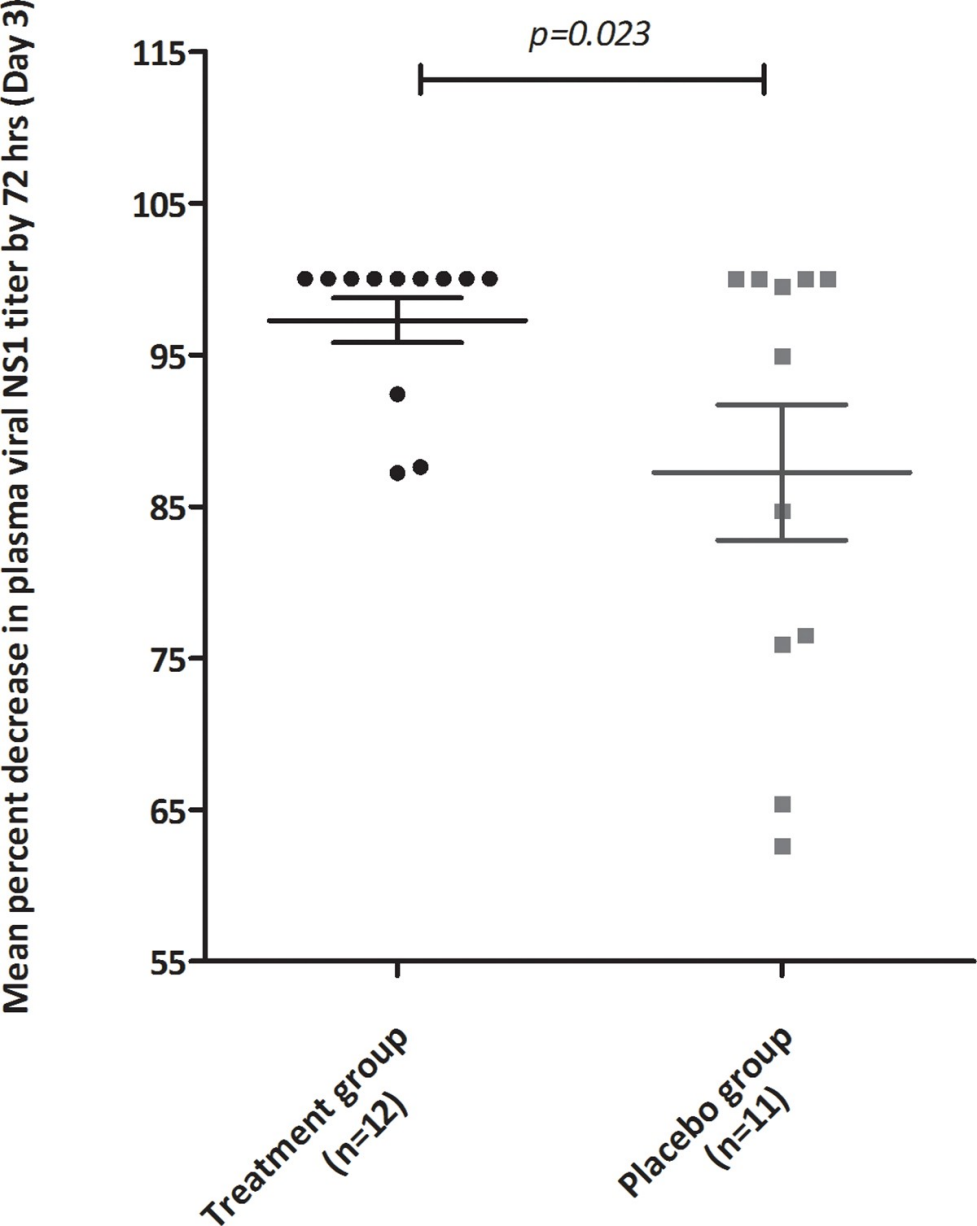
Mean (± s.e.m) percentage decrease in plasma viral NS1 titers by day 3 in treatment group (black) and placebo group (grey) subjects.

NS1 clearance kinetics was evaluated in sub cohort of 26 NS1 antigenemic patients; 16 from treatment and 10 from control group. The pretreatment mean (± s.e.m) plasma levels of NS1 and its clearance kinetics were similar in both groups. The proportions of primary and secondary infections and dengue serotypes in two groups were not significantly different and did not confound the NS1 clearance kinetics. However, by day 3, a significantly (p = 0.023) pronounced mean (± s.e.m) percent decrease in NS1 levels was observed among CPLE patients compared to controls; 97.3% v/s 87.2 resp. **Fig 11.**

#### Adverse events

None of the study patients reported any serious adverse events during the study period. Overall CPLE tablets were safe and well-tolerated.

## Discussion

Although thrombocytopenia is a distinct feature of dengue, there is no direct correlation between the extent of platelet loss and incidences of bleeding manifestations [23,24]. According to the guidelines of NVBDC, Directorate General of Health Services, Ministry of Health & Family Welfare (2008) [25], Government of India, use of platelet transfusions to manage severe thrombocytopenia is not recommended even at a platelet count of <20 × 10^3^/μl. Prophylactic platelet transfusion is advised only when platelets fall below 10 × 10^3^/μl in the absence of bleeding or in prolonged shock coupled with coagulopathy. Despite these recommendations, in reality however, platelet transfusions are frequently administered preemptively to avert bleeding. However, recent studies have demonstrated that risks from such prophylactic transfusions far outweigh any clinical benefit. There is more evidence now to support restrictive platelet transfusions, especially in resource limited settings, to manage bleeding risk in patients with dengue and thrombocytopenia. In such a scenario, availability of a safe, effective and s inexpensive alternative that improves platelet count in dengue infections would be highly beneficial [26,27,28]

Our pilot study findings suggest that CPLE administration is safe and may be efficacious in improving platelet numbers in dengue with severe thrombocytopenia (<30,000/μL). Previous studies evaluating the efficacy of CPLE had demonstrated similar results. In a study cohort of 400 dengue patients with mild to moderate thrombocytopenia, *Ajeeth kumar Gadhwal* et al showed that CPLE treated patients reported a rapid increase in platelet counts and reduced hospitalization days [29]. Similarly, *Kasture* et al [30] also reported an increase in platelet counts to more than 150,000/μL by day 5 in patients administered CPLE compared to only 95,000/μL in the placebo group. Our study demonstrated similar outcomes wherein a significant increase of almost 500% was noted in the platelet counts by day 3. Furthermore, this increase, although insignificant, was faster within treatment group; a median time of only 2 days was observed for platelets to increase to 50,000/μL compared to 3 days in controls. It may be noted that subgroup analysis to adjust for confounder variables especially of NS1 positivity, showed that a significant increase in platelet counts was observed predominantly in study subjects who tested negative for the presence of the viral antigen NS1. As a diagnostic marker of viral infection, dengue NS1 positivity is observed early in the course of infection and our study findings suggest that timing of dengue infection may be an important factor to be considered in efficacy evaluation of CPLE.

Thrombocytopenia in dengue is usually transient with recovery to normal levels occurring naturally in the course of infection. It has been reported that 31% of patients with thrombocytopenia usually present with evidence of active bleeding and the most common site is skin followed by oropharynx [31]. Notably, our study group patients with platelet counts less than 30,000/μL, demonstrated only minor bleeding manifestations like petechiae, purpura, ecchymosis, oropharyngeal/gum bleeding and epistaxis and platelet transfusions were administered to only few (5/50) patients in both groups, especially within the first 48 hours post enrollment. The decisions to transfuse platelets were based on the treating physician’s discretion and the study team did not interfere. Studies [32,33]^,^ have shown that effect of platelet transfusions on platelet counts is transient (<5hrs) and not significant and so, it is highly unlikely that these prophylactically administered platelet transfusions influenced or confounded our findings of an overall positive trend in platelet numbers in the treatment group. Also, by day 3, none of the patients in the treatment group required transfusions whereas one third of the patients in the placebo group continue to be transfusion dependent. Since the normal turnover rate of platelets from megakaryocytes is about 4–6 days, the observed rapid increase in platelet counts (within 72hrs) in the treatment group may be attributable to the megakaryopoietic/thrombopoietic stimulatory activity of CPLE.

The critical phase of dengue is usually when plasma leakage and thrombocytopenia occur, resulting in haemoconcentration. An increase of 20% in HCT values from baseline is an early prognosticator of dengue severity or DHF [34]. The study cohort predominantly consisted of patients in the critical phase of dengue illness and presented with elevated baseline HCT values (>40%). However, CPLE treatment did not significantly alter the kinetics of HCT decrease over the study period and thus did not significantly influence hemoconcentration in the severely thrombocytopenic dengue patients. Additionally, unlike in *Prabhu Kasture et al*’s study, we did not observe any significant elevation of WBCs from the baseline value in the CPLE group [30]. Our analysis also showed that a pronounced increase in platelet counts with CPLE treatment occurred mostly in patients with primary dengue and in their critical phase of illness. Further, our study findings indicate that CPLE treatment modulates the infection induced cytokine levels of TNFα, IFNγ, IL6 and IL4. Lower levels of pro-inflammatory cytokines like TNFα, IFNγ and IL 6 and increased levels of the Th2 cytokine IL4 in the CPLE treated group correlated with increased platelet counts. These findings, although in a small subset, suggest a possible role of CPLE in modulating the underlying infection induced immune response leading thrombocytopenia in dengue infections. Surprisingly, we also observed a significantly faster clearance of NS1 in the treatment group indicating a potential antiviral effect of CPLE. While an antiviral role of CPLE has not yet been described in the literature, few *in-silico* studies have predicted a potential anti-NS2B-NS3 protease activity [35,36]. Nevertheless, this observed effect needs further validation in larger study cohorts as this subgroup analysis was not powered to identify significant antiviral differences.

## Conclusion

Our pilot study demonstrates that while CPLE treatment significantly improves the platelet counts in severe thrombocytopenia in dengue, large prospective studies are required to validate the study findings which are very relevant and cost effective for the resource limited dengue endemic regions.

## Supporting information

S1 Checklist CONSORT 2010 checklist of information to include when reporting a randomised trial*. (DOC)

S1 Appendix STUDY ID Case Report Form (CRF). (DOCX)

S2 Appendix Informed consent. (DOCX)

S3 Appendix (PDF)

S4 Appendix Schedule of events. (DOCX)

S5 Appendix Adverse event form. (DOCX)

S6 Appendix Serious Adverse Event (SAE) report form. (DOCX)

S1 Protocol (DOCX)
