# Supplementary material for: Efficacy & safety of Carica papaya leaf extract (CPLE) in severe thrombocytopenia (≤30,000/μl) in adult dengue – Results of a pilot study
Source: PLoS One. 2020 Feb 19;15(2):e0228699. doi: 10.1371/journal.pone.0228699 (PMC7029881; doi:10.1371/journal.pone.0228699)
Supplement: S1 Appendix — (DOCX) [file pone.0228699.s002.docx]

**APPENDIX – I**

**STUDY ID: Case Report Form (CRF)**

| **Demographics** | | |
| --- | --- | --- |
| **Name: MRD No.** | | |
| Gender: | Age: | Weight: |
| Place: | District: | |
| Education/ Occupation: | Address/Phone No: | |
| Date of admission: | Date of discharge/death: | |
| Travel: Yes No | Details of travel: | |

| **Hospital Stay Details** | |
| --- | --- |
| Date of ward admission: | **Date of discharge (ward)** |
| Date of ICU admission: | **Date of discharge (ICU)** |
| Outcome: Discharged / Expired | |

| **Co-morbidities** | **✓ if Present** |
| --- | --- |
| Type 2 DM |  |
| Systemic HTN |  |
| DLP |  |
| CVA |  |
| CAD |  |
| On Anti platelets (*if yes, day of stoppage*) |  |
| ITP |  |
| CLD |  |
| Past H/o Dengue |  |
| Others (*CKD, Cancer, Connective tissue disorders, On chronic steroids*) |  |

| History of fever: | No of days with fever: | Onset of fever: |
| --- | --- | --- |

| **CLINICAL ASSESSMENT** | | | | | | | |
| --- | --- | --- | --- | --- | --- | --- | --- |
| **Warning signs** | **Day 0** | **Day 1** | **Day 2** | **Day 3** | **Day 4** | **Day 5** | **Day 6** |
| Abdominal pain or tenderness |  |  |  |  |  |  |  |
| Persistent vomiting |  |  |  |  |  |  |  |
| Lethargy |  |  |  |  |  |  |  |
| Restlessness |  |  |  |  |  |  |  |
| Rapid breathing |  |  |  |  |  |  |  |
| Mucosal bleed* |  |  |  |  |  |  |  |
| Liver enlargement > 2cm or tender enlarged liver |  |  |  |  |  |  |  |
| Clinical fluid accumulation & site |  |  |  |  |  |  |  |
| Temperature (°F) |  |  |  |  |  |  |  |
| BP |  |  |  |  |  |  |  |
| Pulse rate |  |  |  |  |  |  |  |
| Respiratory rate |  |  |  |  |  |  |  |

*Mucosal bleed*:- Subcutaneous bleeding, minor mucosal hemorrhages, sub-conjunctival hemorrhage, epistaxis, petechiae, or ecchymosis, positive tourniquet test.*

| **CLINICAL CRITERIA FOR SEVERE DENGUE DIAGNOSIS** | | |
| --- | --- | --- |
| **Signs** | **✓if Present** | **Date of presentation** |
| Severe plasma leakage leading to shock/ fluid accumulation with respiratory distress |  |  |
| Severe bleeding as evaluated by clinician* |  |  |
| LFT (AST or ALT > 1000) |  |  |
| CNS (impaired consciousness) |  |  |
| Cardiac involvement |  |  |
| Other Organ involvement |  |  |

*Severe bleeding as evaluated by clinician*:- internal organ bleeding, hematemesis, intracranial bleed and melena*

| **Days*** | **Day 0** | | **Day 1** | | **Day 2** | | **Day 3** | | **Day 4** | | **Day 5** | | **Day 6** | | **Follow up visit** | **Day 15** | **Day 30** |
| --- | --- | --- | --- | --- | --- | --- | --- | --- | --- | --- | --- | --- | --- | --- | --- | --- | --- |
| **LABORATORY ASSESSMENTS** | | | | | | | | | | | | | | | | | |
| **Hematology** | | | | | | | | | | | | | | |  |  | |
| **Days** | Day 0 | | Day 1 | | Day 2 | | Day 3 | | Day 4 | | Day5 | | Day 6 | |  | Day 15 | Day 30 |
| WBC |  | |  | |  | |  | |  | |  | |  | |  |  | |
| Neutrophils |  | |  | |  | |  | |  | |  | |  | |  |  |  |
| Monocytes |  | |  | |  | |  | |  | |  | |  | |  |  |  |
| Lymphocytes |  | |  | |  | |  | |  | |  | |  | |  |  |  |
| Eosinophils |  | |  | |  | |  | |  | |  | |  | |  |  |  |
| Basophils |  | |  | |  | |  | |  | |  | |  | |  |  |  |
| Platelet |  | |  | |  | |  | |  | |  | |  | |  |  |  |
| RBC |  | |  | |  | |  | |  | |  | |  | |  |  |  |
| Hb |  | |  | |  | |  | |  | |  | |  | |  |  |  |
| HCT (%) |  | |  | |  | |  | |  | |  | |  | |  |  |  |
| MPV |  | |  | |  | |  | |  | |  | |  | |  |  |  |
| RDW |  | |  | |  | |  | |  | |  | |  | |  |  |  |
| **Biochemistry** | | | | | | | | | | | | | | | | | |
| Na+ |  | |  | |  | |  | |  | |  | |  | |  |  | |
| K+ |  | |  | |  | |  | |  | |  | |  | |  |  |  |
| Blood Urea |  | |  | |  | |  | |  | |  | |  | |  |  |  |
| S Creatinine |  | |  | |  |  |  | |  |  |  | |  | |  |  |  |
| S Bilirubin |  | |  | |  |  |  | |  |  |  | |  | |  |  |  |
| SGPT |  | |  | |  |  |  | |  |  |  | |  | |  |  |  |
| SGOT |  | |  | |  |  |  | |  |  |  | |  | |  |  |  |
| ALP |  | |  | |  |  |  | |  |  |  | |  | |  |  |  |
| S Albumin |  | |  | |  |  |  |  |  |  |  | |  | |  |  |  |
| S Globulin |  | |  | |  |  |  |  |  |  |  | |  | |  |  |  |
| S Calcium |  | |  | |  |  |  |  |  |  |  | |  | |  |  |  |
| PT/INR |  | |  | |  |  |  | |  |  |  | |  | |  |  |  |
| APTT |  | |  | |  |  |  | |  |  |  |  |  | |  |  |  |
| URE – ≥6 pus cells/ RBCs/ proteins |  | |  | |  |  |  | |  |  |  |  |  | |  |  |  |
| Trop I |  | |  | |  |  |  |  |  |  |  |  |  | |  |  |  |
|  |  | |  | |  | |  | |  | |  | |  | |  |  |  |
|  |  | |  | |  | |  | |  | |  | |  | |  |  |  |
|  |  | |  | |  | |  | |  | |  | |  | |  |  |  |
|  |  | |  | |  | |  | |  | |  | |  | |  |  |  |
|  |  | |  | |  | |  | |  | |  | |  | |  |  |  |
|  |  | |  | |  | |  | |  | |  | |  | |  |  |  |
| **VIROLOGY LABORATORY ASSESSMENTS** | | | | | | | | | | | | | | | | | |
| **Viral Correlates** | | | | | | | | | | | | | | | | | |
| **Days*** | | **Day 0** | | **Day 1** | **Day 2** | **Day 3** | | **Day 4** | | **Day 5** | | **Day 6** | |  | | | |
| Serotype | |  | |  |  |  | |  | |  | |  | |  |  |  |  |
| Dengue IgM | |  | |  |  |  | |  | |  | |  | |  |  |  |  |
| Dengue IgG | |  | |  |  |  | |  | |  | |  | |  |  |  |  |
| NS1 Antigen | |  | |  |  |  | |  | |  | |  | |  |  |  |  |
| Viremia (*cps/ml*) | |  | |  |  |  | |  | |  | |  | |  |  |  |  |
| **Immune correlates** | | | | | | | | | | | | | | | | | |
| Interleukin 8 | |  | |  |  |  | |  | |  | |  | |  | | | |
| Interleukin 6 | |  | |  |  |  | |  | |  | |  | |  |  |  |  |
| Interleukin 10 | |  | |  |  |  | |  | |  | |  | |  |  |  |  |
| TNF alpha | |  | |  |  |  | |  | |  | |  | |  |  |  |  |
| IFN gamma | |  | |  |  |  | |  | |  | |  | |  |  |  |  |
| IL12p70 | |  | |  |  |  | |  | |  | |  | |  |  |  |  |

* *Days – Day of admission is counted as day 1 and in order to get the phase of dengue fever in which the patient presents, the day has to be counted from the onset of fever.*

| **Treatment/Management** | | | | | | | |
| --- | --- | --- | --- | --- | --- | --- | --- |
| Days^*^ | **Day 0** | **Day 1** | **Day 2** | **Day 3** | **Day 4** | **Day 5** | **Day 6** |
| Crystalloids in ml/day |  |  |  |  |  |  |  |
| Colloids in ml/day |  |  |  |  |  |  |  |
| Blood product and type and quantity (ml) |  |  |  |  |  |  |  |
| Total intake |  |  |  |  |  |  |  |

Signature: _________________________________ Date: ___________________________

Name of Doctor: ______________________________
