# Supplementary material for: Efficacy & safety of Carica papaya leaf extract (CPLE) in severe thrombocytopenia (≤30,000/μl) in adult dengue – Results of a pilot study
Source: PLoS One. 2020 Feb 19;15(2):e0228699. doi: 10.1371/journal.pone.0228699 (PMC7029881; doi:10.1371/journal.pone.0228699)
Supplement: S2 Appendix — (DOCX) [file pone.0228699.s003.docx]

**APPENDIX –II (English)**

INFORMED CONSENT

**Introduction:**

**Invitation and Purpose of the study:**

We are conducting a study in which we are trying to understand the effectiveness of treating severe thrombocytopenia (< 30K/µl) in dengue infections with oral Caripill tablets (1100mg t.i.d) for 5 days.

With your permission, we would like to conduct a placebo-controlled, single centre, randomised prospective study of Caripill (Carica papaya leaf extract) tabs in dengue patients with moderate to severe thrombocytopenia (platelets < 50K/ul) . The study will enrol 100 eligible dengue cases into the two arms of the study – one arm receiving Caripill 1100mg t.i.d for 5 days and the other group receiving placebo.

Please go through the details of the project below (details to be mentioned)

| **Title:** | A propsective study to evaluate the safety and efficacy of Carica papaya leaf extract (oral caripill) in the treatment of severe thrombocytopenia (<30,000/µl) in dengue |
| --- | --- |
| **Précis:** | This will be a placebo-controlled, single center, randomized prospective study of Caripill (*Carica papaya* leaf extract) tabs in dengue patients with severe thrombocytopenia (platelets < 30K/ul). The study will enrol 100 eligible dengue cases into the two arms of the study – one arm receiving Caripill 1100mg *t.i.d* for 5 days and the other group receiving placebo. |
| **Objectives:** | Primary: To determine the impact of Caripill administration (1100mg t.i.d for 5 days on the following:   - Platelet count and hematocrit values in dengue patients with severe thrombocytopenia. - Reducing the number of blood product transfusions esp. platelet transfusion and - All-cause mortality rates. |
|  | Secondary: To evaluate the effect of Caripill administration (1100mg t.i.d for 5 days) on the following-   - Magnitude and kinetics of serum viremia - Magnitude and kinetics of cytokines IL12p70, IFNγ, IL6, TNFα and IL8. - The total length of hospital (ICU & ward) stay, - Total cost of care. |
| **Population:** | Study cohort of 100 adults, either gender (more than18 yrs old, non-pregnant) and admitted to AIMS Hospital and diagnosed of dengue with moderate to severe thrombocytopenia. |
| **Number of Sites:** | Dept. of General Medicine and Dept. of Emergency Medicine, Amrita Institute of Medical Sciences and Research Center, Ponekkara, AIMS, Kochi-682041. |
| **Description of Intervention:** | Orally available Caripill Tabs (MicroLab) at a dose of 1100mg/tab three times a day for 5 days |
| **Study Duration:** | The estimated time (in months) from when the study opens to enrollment until completion of data analyses will be 12 months. |
| **Subject Participation Duration:** | Duration of hospital stay and the subsequent two follow-ups at 2 and 4 weeks post last dose of Caripill. |
| **Estimated Time to Complete Enrollment:** | Immediately after screening positive and signing the informed consent form (ICF). |

**Do I have to take part in this study**

Your participation in this study is voluntary. It is up to you to decide whether to participate in this study. If you change your mind, you are free to withdraw from the study at any time and without giving a reason. Your decision to participate or not will not affect the standard of care you /your child receive. You can withdraw from the study at any time and refusal to participate will not involve any penalty or loss.   You will receive the standard of care treatment you are already eligible for.

**What do I have to do in this study? What is being studied?**

If You agree to participate in this study you will be required to take the Caripill tablets or placebo three times a day for 5 days and agree to visit or telephonically followed up at 2 week and 4 week intervals after last dose admininstered.

**What are the Methods employed in the study?**

Routinue biochemical, haematological and microbiological testing of peripheral blood will be done. About 5 -10ml of blood will be collected in different tubes and send to the Biochemistry, Hematology and Virology Labs for analysis. The blood may also be used for future research studies including genetic analysis, if required.

**What are the risks in taking part in this study?**

No major risks are involved in taking part in this study

**What are the Benefits?**

There are important benefits of the proposed Caripil treatment on the study population. A number of studies have demonstrated the effectiveness of Caripill in increasing the total as well as rate of platelet counts and hematocrit levels in dengue patients with mild to moderate. Give the high risk of hemorrhages and shock in severe dengue cases and its associated mortality, any potential benefit of improvement in thrombocytopenia will be highly beneficial.

**Is participation compulsory?**

No. Taking part in this study is on a voluntary basis. If you have any doubt please do not hesitate to ask.

**How will the information collected in the study be used?**

The information collected will be kept completely confidential.  At no point will we reveal the identity of the individual patients when we analyze the results. Your information will be archived in a coded form. This will not include name, address or any other private information. Your   permission to the study doctor and staff to use this information does not automatically end at a particular time.

However your details will be released to any regulatory authorities  if requested for legal/auditing purpose.

**Problems or Questions**:

If you have any question about the study, you may contact your doctor, whose details will be provided to you. You will also be asked to provide your address and contact details in case if we need to contact you during the study.

Name and Contact Details of the Investigator:

Name and contact details of local/Central ethics committee chairperson/member

I,……………………………………………(Name of the Patient) undergoing treatment in the **Amrita Institute of Medical Sciences,  Kochi** with MRD No:…………… hereby affirm and state as follows: -

My physician  Dr.Vidya P.Menon, after examining me and conducting the necessary tests has diagnosed my condition as (Provisional diagnosis) dengue with severe thromobocytopenia and has sought my consent to participate in the study .

   I confirm that I have read the written information for study, “A PROPSECTIVE STUDY TO EVALUATE THE SAFETY AND EFFICACY OF Carica papaya LEAF EXTRACT (ORAL CARIPILL) IN THE TREATMENT OF SEVERE THROMBOCYTOPENIA (<30,000/µl) IN DENGUE”, and confirm that I have had the opportunity to ask questions about this study and I am satisfied with the answers and explanations that have been provided.

•     I understand that I grant access to data to authorized persons described in the information sheet

•     have been given time and opportunity to consider taking part in this study.

•     I understand my participation is voluntary and that I am free to withdraw at any time, without giving reason

•     I agree to take part in this study.

I confirm that I have been informed by  my treating doctor that  my clinical data  , diagnostic reports / films, any photographs or video graphs etc will be  used for presentation /scientific publication and research analysis . I understand that such use  is for  advancement of  further medical knowledge. I voluntarily agree to such  use  . I also understand that my identity will not  be revealed in such presentation I express my willingness to the same . Having understood all the facts and the conditions stated above  I accept and agree and hereby express my free and voluntary consent to  Dr. Vidya P.Menon and her associates to participate in the study .

I understand that I have not given up any of my rights by signing this form. I will receive the full copy of the informed consent referred to above, including this signed statement with my signature below.

Signature of patient: Signature of Father/Mother/Legal Name of patient:

Guardian:

Address: Address:

Date: Date:

Qualification and Occupation of the subject

Annual Income of the subject

Name and Address of the Nominee

Signature of witness: Signature of witness:

Name of witness: Name of witness:

Address: Address:

Date: Date:

 I have explained and made Mr/Mrs .................................understand the above mentioned details of the study. -

Signature:

Name:

Address:

Date:
