# Supplementary material for: Efficacy & safety of Carica papaya leaf extract (CPLE) in severe thrombocytopenia (≤30,000/μl) in adult dengue – Results of a pilot study
Source: PLoS One. 2020 Feb 19;15(2):e0228699. doi: 10.1371/journal.pone.0228699 (PMC7029881; doi:10.1371/journal.pone.0228699)
Supplement: S3 Appendix — (PDF) [file pone.0228699.s004.pdf]

Appendix II

ധാരണാപത്രിക

ആമുഖം

ഇന്ന് ലോകമെമ്പാടും നേരിടുന്ന ഒരു ഗൗരവമായ പ്രശ്നമാണ് ആന്റിബയോട്ടിക്കിനെതിരെ പ്രതിരോധിക്കുന്ന അണുക്കളുടെ വർധനവും അതുമൂലം ഉണ്ടാകുന്ന അണുബാധയും. പ്രേത്യേകിച്ചു ഐ.സി.ഉ ഇൽ കിടക്കുന്ന ഗുരുതരമായ രോഗികളിൽ ഇപ്പോൾ ലഭ്യമായ ആന്റിബിയോട്ടിക്സ്, ഈ അണുക്കൾക്കെതിരെ പ്രവർത്തിക്കാത്തതിനാൽ, കൊളിസ്റ്റിൻ എന്ന ആന്റിബൈക്കോടിക് ഉപയോഗം വർധിച്ചു. എന്നാൽ കൊളിസ്റ്റിന് വൃക്കയിൽ ഉണ്ടാകുന്ന ദൂഷ്യ ഫലം കാരണം ഇത് സാധാരമായി ഉപയോഗിക്കാറില്ല. ഈ ആന്റിബയോട്ടിക്കിന്റെ അളവ് കുറയാനോ കൂടാനോ പാടില്ല, അതായത് ദൂഷ്യഫലം ഉണ്ടാകാത്തതും ചികിത്സക്ക് ഉപയോഗിക്കുന്നതും ആയ അളവ് വേണം ശരീരത്തിൽ എത്താൻ . അതിനാൽ കൊളിസ്റ്റിന്റെ അളവ് ഓരോ രോഗിയുടെ വൃക്കയുടെ പ്രവർത്തനം അനുസരിച്ചുള്ള അളവിൽ കൊടുക്കേണ്ടത് ആണ് . എന്നാൽ ഇന്ത്യയിൽ ഉള്ള ജനങ്ങൾക്ക് എത്ര അളവിൽ ആണ് കൊളിസ്റ്റിൻ നൽകേണ്ടതെന്ന് ഉള്ളതിന് ഇതുവരെ നിലവാരമായ നിർദേശം ഇല്ല. ഇന്നും വിദേശ ജനങ്ങളിൽ നടത്തിയ പഠനങ്ങൾ അനുസരിച്ചാണ് ഇവിടെ കൊളിസ്റ്റിൻ അളവ് നിർദേശിച്ചിരിക്കുന്നത്. ഇത് മെച്ചപ്പെടുത്താൻ ഉള്ള ഒരു മാർഗം ആണ് തെറാപ്യൂട്ടിക്ക് ഡ്രഗ് മോണിറ്ററിങ്. ഇത് ഉപയോഗിച്ച രക്തത്തിൽ ഉള്ള കൊളിസ്റ്റിന്റെ അളവ് അളക്കുകയും ,അത് മൂലം നമ്മൾ കൊടുക്കുന്ന കൊളിസ്റ്റിന്റെ അളവ് കുറവോ കൂടുതലോ അല്ല എന്ന് ഉറപ്പു വരുത്താം. ഐ.സി.ഉ ഇൽ കിടക്കുന്ന ഗുരുതരമായ രോഗികളിൽ കോളിസ്റ്റിന് ന്റെ അളവ് വൃക്കക്ക് ദൂഷ്യഫലങ്ങൾ ഉണ്ടാക്കുന്നതല്ല എന്ന് നിരന്തരം നിരീക്ഷിക്കുന്നത് അത്യാവശ്യം ആണ്. അതിനാൽ രക്തത്തിൽ കൊളിസ്റ്റിന്റെ കൃത്യമായ അളവ് ശരീരത്തിൽ ലഭിക്കുക എന്ന് ഉള്ളത് വളരെ ആവശ്യം ആണ്.

പഠനത്തിലേക്കുള്ള ക്ഷണവും പഠനത്തിന്റെ ഉദ്ദേശവും

ഞങ്ങൾ ഈ പഠനം നടത്തുന്നത് ഗുരുതരമായി കിടക്കുന്ന രോഗികളുടെ രക്തത്തിൽ കൊളിസ്റ്റിന്റെ അളവ് കണ്ടുപിടിക്കുകയും അത് ഉപയോഗിച്ച കൊളിസ്റ്റിന്റെ അളവ് കൃത്യമാണോ എന്ന് കണ്ടുപിടിക്കുകയും ആണ് ചെയ്യാൻ ഉദ്ദേശിച്ചിരിക്കുന്നത് . വൃക്ക സാധാരണയായി പ്രവർത്തിക്കുന്ന ഗുരുതരമായ രോഗികളിൽ, ഡോക്ടറിന്റെ നിർദേശപ്രകാരം ഹോസ്പിറ്റൽ മാർഗ്ഗ രേഖ അനുസരിച്ചാകും കൊളിസ്റ്റിൻ നല്കുന്നത്. എന്നാൽ ഇന്ത്യയിൽ ഉള്ള ജനങ്ങൾക്ക് എത്ര അളവിൽ ആണ് കൊളിസ്റ്റിൻ നൽകേണ്ടതെന്ന് ഉള്ളതിന് ഇതുവരെ നിലവാരമായ നിർദേശം ഇല്ല. ഈ പഠനം, നമ്മുടെ നാട്ടിലെ

ഗുരുതരമായി കിടക്കുന്ന രോഗികളിൽ കൊളിസ്റ്റിന്റെ അളവ് നിർദേശിക്കുന്ന നിലവാരം ഉള്ള മാർഗ്ഗരേഖ വികസിപ്പിക്കാൻ സഹായിക്കും.

ഞാൻ ഈ പഠനത്തിൽ പങ്കെടുക്കേണ്ട ആവശ്യം ഉണ്ടോ ?

ഈ പഠനത്തിൽ താങ്കളുടെ പങ്കാളിത്തം തികച്ചും സ്വന്തം ഇഷ്ടപ്രകാരമാണ്. ഈ പഠനത്തിൽ പങ്കെടുക്കണോ എന്ന് ഉള്ളത് താങ്കൾക്ക് തീരുമാനിക്കാം. പഠനം തുടങ്ങിയ ശേഷം, എപ്പോഴെങ്കിലും താങ്കളുടെ തീരുമാനം മാറ്റുകയാണെങ്കിൽ, എപ്പോ വേണമെങ്കിലും ഒരു കാരണവും ഇല്ല്ലാതെ താങ്കൾക്ക് ഈ പഠനത്തിൽ നിന്ന് പിൻവാങ്ങാൻ, ഈ തീരുമാനം താങ്കൾക്ക് ലഭിക്കുന്ന ചികിത്സയെ ഒരു രീതിയിലും സ്വാധീനിക്കുന്നതല്ല . എപ്പോ വേണമെങ്കിലും ഈ പഠനത്തിൽ നിന്ന് താങ്കൾക്ക് പിൻവാങ്ങാൻ സാധിക്കും. അത് മൂലം താങ്കൾക്ക് യാതൊരു രീതിയിലും ഉള്ള നഷ്ടം സംഭവിക്കില്ല. അർഹമായ എല്ലാ ചികിത്സയും താങ്കൾക്ക് ലഭിക്കും.

ഞാൻ ഈ പഠനത്തിൽ എന്താണ് ചെയേണ്ടത്? എന്താണ് ഇതിൽ പഠിക്കുന്നത്?

താങ്കൾ ഈ പഠനത്തിൽ പങ്കെടുക്കാൻ സമ്മതിച്ചാൽ താങ്കൾക്ക് കൊളിസ്റ്റിൻ ആദ്യം 9 എം യു ഇൻജെക്ഷൻ ഒറ്റ തവണയും തുടർന്ന് 3 എം യു ഇൻജെക്ഷൻ ദിവസേന മൂന്ന് പ്രാവശ്യവും ലഭിക്കുന്നതാണ്. ഈ തരുന്ന മരുന്ന് താങ്കളുടെ രക്തത്തിൽ കൃത്യമായ അളവിൽ ലഭിക്കുന്നുണ്ടോ എന്നാണ് ഈ പഠനം കണ്ടുപിടിക്കാൻ ശ്രമിക്കുന്നത് .

എന്ത് മാർഗം ആണ് ഈ പഠനത്തിൽ ഉപയോഗിച്ചിരിക്കുന്നത് ?

താങ്കളുടെ രക്തത്തിന്റെ ഒരു ചെറിയ അളവ്, അതായത് 3 മില്ലി , ആദ്യത്തെ മരുന്ന് നൽകുന്നതിന് മുന്പും , ആ മരുന്ന് നൽകിയതിനു ശേഷം 0.5,1,2,4,8,12 മണിക്കൂറുകളി ലും എടുക്കുന്നതാണ്. ഇതേ പോലെ താങ്കൾക്ക് ലഭിക്കുന്ന എട്ടാമത്തെ ഡോസിന് തൊട്ടു മുമ്പും ഒരു മണിക്കൂർ, രണ്ടു മണിക്കൂർ, എട്ടു മണിക്കൂർ കൊളിസ്റ്റിൻ ഡോസ് കൊടുത്ത ശേഷവും എടുക്കുന്നത് ആണ്. ഇതേ പോലെ ഒൻമ്പതാമത്തെ ഡോസിന് തൊട്ടു മുന്പും, ഡോസ് കൊടുത്ത ശേഷം, ഒരു മണിക്കൂർ, രണ്ടു മണിക്കൂർ എട്ടു മണിക്കൂർ ശേഷവും രക്തം എടുക്കുന്നതാണ്.ഓരോ രോഗിയിൽ നിന്നും 14 തവണ രക്തത്തിന്റെ സാമ്പ്ൾസ് എടുക്കുന്നതാണ്.

ഇതിൽ പങ്കെടുക്കുന്നത് മൂലം എന്തൊക്കെ റിസ്ക് ആണ് എനിക്ക് ഉള്ളത് ?

കൊളിസ്റ്റിൻ വൃക്കയിൽ ദൂഷ്യഫലങ്ങൾ ഉണ്ടാകുന്നതായി പഠനങ്ങൾ സ്ഥിദ്ധീകരിച്ചിട്ടുണ്ട് . എന്നാൽ ഈ പഠനത്തിൽ , ഓരോ രോഗിയുടെയും വൃക്കയുടെ പ്രവർത്തനം നിരീക്ഷിക്കുകയും അതിനു അനുസൃതമായ ഡോസിൽ മാറ്റങ്ങൾ ചെയ്യുന്നതാണ്. അതെ പോലെ തങ്ങളുടെ ഡോക്ടറിന്റെ

നിർദേശപ്രകാരം വൃക്കയുടെ പ്രവർത്തനം ശരിയാക്കാനുള്ള കാര്യങ്ങളും ചെയുന്നതാണ്

എന്തൊക്കെ ആണ് ഗുണങ്ങൾ ?

രക്തത്തിൽ കൊളിസ്റ്റിന്റെ അളവ് വിവിധ സമയങ്ങളിൽ കണ്ടുപിടിച്ചാൽ , അത് ഉപയോഗിച്ച ഇന്ത്യയിലെ രോഗികൾക്ക് രോഗ ചികിത്സക്ക് ആവശ്യമായ ഡോസിൽ ആണോ കോളിസ്റ്റിന് കൊടുക്കുന്നതെന്ന് ഉറപ്പുവരുത്താൻ സാധിക്കും. അതല്ല എന്ന് ഉണ്ടെങ്കിൽ ഇന്ത്യയിലെ ജനങ്ങളിൽ കൊളിസ്റ്റിൻ ഡോസിന്റെ ഒരു നിലവാരമുള്ള ഔഷധവിധി വികസിപ്പിക്കാൻ ഈ പഠനം മൂലം സാധിക്കും.

ഇതിൽ പങ്കെടുക്കേണ്ടത് നിർബന്ധം ആണോ?

ഈ പഠനത്തിൽ പങ്കെടുക്കുകയോ വേണ്ടയോ എന്ന് ഉള്ളത് താങ്കളുടെ താല്പര്യം ആണ്. എന്ത് സംശയമുണ്ടെങ്കിലും ഒരു മടിയും കൂടാതെ താങ്കൾക്ക് ചോദിക്കാം.

ഈ പഠനത്തിനായി ശേഖരിക്കുന്ന വിവരങ്ങൾ എങ്ങനെ ഉപയോഗിക്കും?

ഈ പഠനത്തിൽ നിന്ന് ലഭിക്കുന്ന എല്ലാ വിവരങ്ങളും സ്വകാര്യമായി സൂക്ഷിക്കുന്നതാണ്. എല്ലാ വിവരങ്ങളും ഒരു കോഡ് രൂപത്തിൽ ആകും സൂക്ഷിക്കുക. അതിൽ താങ്കളുടെ പേരോ, വിലാസമോ മറ്റു സ്വകാര്യ വിവരങ്ങളോ ഉൾ പ്പെടുത്തുന്നതല്ല . എന്നാൽ പഠന സംബന്ധമായ വിവരങ്ങൾ റെഗുലേറ്ററി അഥവാ എത്തിക്കൽ കമ്മിറ്റിയിലേക്ക് സമർപ്പിക്കുന്നതാണ്.

പ്രശ്നങ്ങളോ ച്യോദ്യങ്ങളോ

പഠന സംബന്ധമായി എന്ത് സംശയവും താങ്കൾക്ക് ഡോക്ടറിനോട് ചോദിക്കാവുന്നതാണ്. ഡോക്ടറിനെ ബന്ധപ്പെടാനുള്ള വിവരങ്ങൾ താങ്കൾക്ക് നൽകുന്നതായിരിക്കും . പഠനവേളയിൽ താങ്കളുമായി ബന്ധപെടാനുള്ള വിലാസവും മറ്റു വിവരങ്ങളും നൽകേണ്ടതാണ്.

ഇൻവെസ്റിഗേറ്ററിന്റെ പേര് : Dr വിദ്യ പി മേനോൻ

ലോക്കൽ / സെൻട്രൽ എത്തിക്സ് കമ്മിറ്റി ചെയർപേഴ്സൺ / മെമ്പറിന്റെ പേര് :

അമൃത ഇൻസ്റ്റിറ്റ്യൂട്ട് ഓഫ് മെഡിക്കൽ സയൻസിൽ ചികിത്സയിൽ കഴിയുന്ന
_____________________ എന്ന് പേരായ _____________ എം.ർ.ഡി നമ്പർ
ആയ ഞാൻ സ്വമനസാലെ നൽകുന്ന സമ്മതപാത്രം.

ഞാൻ മുകളിൽ പറഞ്ഞതിരിക്കുന്ന വിവരങ്ങൾ വായിച്ചുമനസിലാക്കിയിട്ടുണ്ട്.

ഈ പഠനസംബന്ധമായി എനിക്ക് സംശയങ്ങളോ ചോദ്യങ്ങളോ ചോദിക്കാനുള്ള അവസരം ലഭിച്ചിട്ടുണ്ട്. എനിക്ക് ലഭ്യമായിട്ടുള്ള ഉത്തരങ്ങൾ എനിക്ക് ഏറെ സംതൃപ്തിയുണ്ട്. എന്റെ പഠനപങ്കാളിത്ത സ്വമേധയാ ആണ്, എന്ന് ഞാൻ മനസ്സിലാക്കുന്നു. ഈ പഠനത്തിൽ നിന്നും എപ്പോൾ വേണമെങ്കിലും പിന്മാറാൻ സാധിക്കുമെന്ന് ഞാൻ മനസ്സിലാക്കുന്നു. പിന്മാറുന്നതിനു മറ്റു വിശദീകര ണങ്ങൾ നൽകേണ്ടതില്ല എന്നും ഞാൻ മനസിലാകുന്നു. എന്റെ പഠന റെക്കോർഡുകളും ചികിത്സ രേഖകളും പരിശോധിക്കുന്നതിനായി ഞാൻ ഡോക്ടർ ക്കു പൂർണ സമ്മതം നൽകുന്നു. എന്റെ പങ്കാളിത്തം തികച്ചും സ്വമേധയാണ്.

ഞാൻ മുകളിൽ പറഞ്ഞിരിക്കുന്ന വിവരങ്ങൾ വായിച്ചുമനസ്സിലാക്കിയിട്ടുണ്ട്.

ഈ പഠനസംബന്ധമായി എനിക്ക് സംശയങ്ങളോ ചോദ്യങ്ങളോ ചോദിക്കാനുള്ള അവസരം ലഭിച്ചിട്ടുണ്ട്. എനിക്ക് ലഭ്യമായിട്ടുള്ള ഉത്തരങ്ങൾ എനിക്ക് ഏറെ സംതൃപ്തിയുണ്ട്. എന്റെ പഠനപങ്കാളിത്തതം സ്വമേധയാ ആണ്, എന്ന് ഞാൻ മനസിലാകുന്നു. ഈ പഠനത്തിൽ നിന്നും എപ്പോൾ വേണമെങ്കിലും പിന്മാറാൻ സാധിക്കുമെന്ന് ഞാൻ മനസ്സിലാക്കുന്നു. പിന്മാറുന്നതിനു മറ്റു വിശദീകരങ്ങൾ നൽകേണ്ടതില്ല എന്നും ഞാൻ മനസിലാകുന്നു. എന്റെ പഠന റെക്കോർഡുകളും ചികിത്സ രേഖകളും പരിശോധിക്കുന്നതിനായി ഞാൻ ഡോക്ടർ ക്കു പൂർണ സമ്മതം നൽകുന്നു. എന്റെ പങ്കാളിത്തം തികച്ചും സ്വമേധയാ സ്വമേധയാണ്.

എന്റെ പഠന സംബന്ധമായ വിവരങ്ങൾ ശാസ്ത്രപഠനത്തിന്റെ ഭാഗമായിട്ട് നടത്തുന്ന അവതരണങ്ങൾക്കും പ്രസിദ്ധീകരണത്തിനു മറ്റും ഉപയോഗപ്പെടുത്തുന്നതിന് ഞാൻ സ്വമേധയാ സമ്മതം നൽകുന്നു.

എന്റെ അടിസ്ഥാന വിവരങ്ങളോ മറ്റു സ്വകാര്യവിവരങ്ങളോ പ്രസ്തുത അവതരണങ്ങളിലോ പ്രസിദ്ധീകരങ്ങളിലോ ഉപയോഗിക്കില്ല എന്നും ഞാൻ മനസിലാകുന്നു. ഈ സമ്മതപത്രം നല്കുന്നതുകൊണ്ട് എന്റെ നിയമപ്രകാരം ഉള്ള മറ്റു അവകാശങ്ങൾ ഒന്നുംതന്നെ നഷ്ടപ്പെടുക ഇല്ലെന്നും ഞാൻ മനസിലാകുന്നു.

രോഗിയുടെ യോഗ്യതയും ജോലിയും:

രോഗിയുടെ വർഷ വരുമാനം:

രോഗിയുടെ ഒപ്പ് :
വിലാസം:

തിയതി:

അച്ഛനോ/അമ്മയോ/രക്ഷിതാവിന്റെയോ ഒപ്പ് :
വിലാസം:

തിയതി:

സാക്ഷിയുടെ ഒപ്പ്
വിലാസം

തിയതി:

സാക്ഷിയുടെ ഒപ്പ്
വിലാസം

തിയതി:
