# Supplementary material for: Efficacy & safety of Carica papaya leaf extract (CPLE) in severe thrombocytopenia (≤30,000/μl) in adult dengue – Results of a pilot study
Source: PLoS One. 2020 Feb 19;15(2):e0228699. doi: 10.1371/journal.pone.0228699 (PMC7029881; doi:10.1371/journal.pone.0228699)
Supplement: S4 Appendix — (DOCX) [file pone.0228699.s005.docx]

APPENDIX IV: SCHEDULE OF EVENTS

| Procedures | | Screening  (Day –2 to –0) | | Baseline  (Day 0) | | Study Day 1  (Day 1 ± 0.5) | Study Day 2  (Day 2 ± 0.5) | | Study Day3 3  (Day 3 ± 0.5) | | Study Day4  (Day 4 ± 0.5) | | Study Completion  (Day 5± 0.5) | | | Premature Discontinuation | |
| --- | --- | --- | --- | --- | --- | --- | --- | --- | --- | --- | --- | --- | --- | --- | --- | --- | --- |
| Signed Consent Form | | X | | X | |  |  | |  | |  | |  | | |  | |
| Assessment of Eligibility Criteria | | X | | X | |  |  | |  | |  | |  | | |  | |
| Review of Fever History | | X | | X | |  |  | |  | |  | |  | | |  | |
| Review of Concomitant Medications | | X | | X | | X | X | | X | | X | | X | | | X | |
| Study Intervention | |  | | X | | X | X | | X | | X | |  | | |  | |
| Physical Examination | Complete exam | X |  | |  | | |  | |  | |  | | X | X | |  |
|  | Symptom-Directed |  | X | | (X) | | | (X) | | (X) | | (X) | |  |  | |  |
|  | Vital Signs |  | (X) | | (X) | | | (X) | | (X) | | (X) | |  |  | |  |
| Behavioral Assessment | | X | | X | |  |  | |  | |  | | X | | |  | |
| Assessment of Adverse Events | |  | |  | | (X) | (X) | | (X) | | (X) | | X | | | X | |
| Clinical Laboratory | Biochemical | X | X | | (X) | | | (X) | | (X) | | (X) | | X | X | |  |
|  | Hematology | X | X | | (X) | | | (X) | | (X) | | (X) | | X | X | |  |
|  | Urinalysis | X | X | | (X) | | | (X) | | (X) | | (X) | | X | X | |  |
| Virology Laboratory | Immunology _5mL whole blood |  | | X | |  | (X) | |  | | (X) | | X | | | X | |
|  | Virology  5 ml EDTA Blood |  | | X | |  |  | |  | |  | | X | | |  | |
|  | Sample for Genetic Analysis |  | | X | |  |  | |  | |  | | X | | |  | |
| Other Procedures |  |  | | (X) | |  | (X) | |  | | (X) | | (X) | | | (X) | |
|  |  |  | |  | |  |  | |  | |  | |  | | |  | |

(X) – Will be performed.

Note: A list of Clinical Laboratory tests, e.g.:

- **Pregnancy Test** – urine or serum test to establish eligibility
- **Hematology** – Hemoglobin, hematocrit, WBC and differential count, platelet count.
- **Biochemistry** – Sodium, potassium, chloride, urea, creatinine, glucose, uric acid, bicarbonate, amylase, lipase, albumin, total bilirubin, cholesterol, triglycerides, and creatine phosphokinase, as appropriate for the study.
- **Urinalysis** (protein and glucose), as appropriate for the study.

List of Research Laboratory tests and the required specimen types, e.g.:

- **Virology -** 5ml of blood (serotype specific PCR, quantitative dengue specific PCR (viral load cps/ml)),**.**
- **Immunology –** 5-10ml of blood.Dengue IgM, IgG quantitative ELISA, cytokine ELISAs.
- **Gene sequencing -** 1mL blood. DNA extraction,
