# Supplementary material for: Efficacy & safety of Carica papaya leaf extract (CPLE) in severe thrombocytopenia (≤30,000/μl) in adult dengue – Results of a pilot study
Source: PLoS One. 2020 Feb 19;15(2):e0228699. doi: 10.1371/journal.pone.0228699 (PMC7029881; doi:10.1371/journal.pone.0228699)
Supplement: S5 Appendix — (DOCX) [file pone.0228699.s006.docx]

| **A PROPSECTIVE STUDY TO EVALUATE THE SAFETY AND EFFICACY OF *Carica papaya* LEAF EXTRACT (ORAL CARIPILL) IN THE TREATMENT OF SEVERE THROMBOCYTOPENIA (<30,000/µl) IN DENGUE** | | | |
| --- | --- | --- | --- |
| **Site Number:** | ______________________ | **Pt_ID:** | _____________________ |

**Has the participant had any Adverse Events during this study? Yes  No *(If yes, please list all Adverse Events below)***

| **Severity** | **Study Intervention Relationship** | **Action Taken Regarding Study Intervention** | **Outcome of AE** | **Expected** | **Serious** |
| --- | --- | --- | --- | --- | --- |
| 1 = Mild  2 = Moderate  3 = Severe | 1 = Definitely related  2 = Possibly related  3 = Not related | 1 = None  2 = Discontinued permanently  3 = Discontinued temporarily  4 = Reduced Dose  5 = Increased Dose  6 = Delayed Dose | 1 = Resolved, No Sequel  2 = AE still present- no treatment  3 = AE still present-being treated  4 = Residual effects present-not treated  5 = Residual effects present- treated  6 = Death  7 = Unknown | 1 = Yes  2 = No | 1 = Yes  2 = No  (If yes, complete SAE form) |

| Adverse Event | Start Date | Stop Date | Severity | Relationship to Study Treatment | Action Taken | Outcome  of AE | Expected? | Serious Adverse Event? | Initials |
| --- | --- | --- | --- | --- | --- | --- | --- | --- | --- |
| **1.** |  |  |  |  |  |  |  |  |  |
| **2.** |  |  |  |  |  |  |  |  |  |
| **3.** |  |  |  |  |  |  |  |  |  |
