# Supplementary material for: Efficacy & safety of Carica papaya leaf extract (CPLE) in severe thrombocytopenia (≤30,000/μl) in adult dengue – Results of a pilot study
Source: PLoS One. 2020 Feb 19;15(2):e0228699. doi: 10.1371/journal.pone.0228699 (PMC7029881; doi:10.1371/journal.pone.0228699)
Supplement: S6 Appendix — (DOCX) [file pone.0228699.s007.docx]

| Protocol Title: A prospective study to evaluate the safety and efficacy of Carica papaya leaf extract (oral caripill) in the treatment of severe thrombocytopenia (<30,000/µl) in dengue. |
| --- |
| Protocol Number: |
| Pt_ID: |

1. SAE Onset Date: (dd/mmm/yyyy)
2. SAE Stop Date: (dd/mmm/yyyy)
3. Location of serious adverse event (e.g. at study site or elsewhere):
4. Was this an unexpected adverse event?

| Yes | No |
| --- | --- |

1. Brief description of participant with no personal identifiers:

| Sex: | Female | Male | Age: |
| --- | --- | --- | --- |

1. Adverse Event Term(s):
2. Brief description of the nature of the serious adverse event (attach description if more space needed):
3. Category of the serious adverse event:

| death – date (dd/mmm/yyyy) | congenital anomaly / birth defect |
| --- | --- |
| life-threatening | required intervention to prevent |
| hospitalization - initial or prolonged | permanent impairment |
| disability / incapacity | other: |

1. Intervention type:

Medication or Nutritional Supplement: specify

Device: Specify:

Surgery: Specify:

Behavioral/Life Style: Specify:

1. Relationship of event to intervention:

Unrelated (clearly not related to the intervention)

Possible (may be related to intervention)

Definite (clearly related to intervention)

| Yes | No |
| --- | --- |

1. Was study intervention discontinued due to event?
2. What medications or other steps were taken to treat serious adverse event?
3. List any relevant tests, laboratory data, history, including preexisting medical conditions
4. Type of report:

Initial

Follow-up

Final

Signature of Principal Investigator:

Date: (dd/mmm/yyyy)
