# Supplementary material for: Efficacy & safety of Carica papaya leaf extract (CPLE) in severe thrombocytopenia (≤30,000/μl) in adult dengue – Results of a pilot study
Source: PLoS One. 2020 Feb 19;15(2):e0228699. doi: 10.1371/journal.pone.0228699 (PMC7029881; doi:10.1371/journal.pone.0228699)
Supplement: S1 Protocol — (DOCX) [file pone.0228699.s008.docx]

**A PROPSECTIVE STUDY TO EVALUATE THE SAFETY AND EFFICACY OF *Carica papaya* LEAF EXTRACT (ORAL CPLE (CARIPILL)) IN THE TREATMENT OF SEVERE THROMBOCYTOPENIA (**≤**30,000/µl) IN DENGUE**

**Principal Investigator:**

**Dr.Vidya P.Menon MD, DNB, FACP**

**Industry Sponsor: Micro Labs Limited, Bangalore, India.**

**Funded by: Micro Labs Limited, Bangalore, India**

**8th August 2016**

TABLE OF CONTENTS

PAGE

[TABLE OF CONTENTS 2](#_Toc456962013)

[LIST OF ABBREVIATIONS 4](#_Toc456962014)

[PROTOCOL SUMMARY 5](#_Toc456962015)

STUDY SCHEMA .….………..…………………………………………….……………………………………………………………6

[1 KEY ROLES AND CONTACT INFORMATION……….…………..…………………………………………………...7](#_Toc456962016),8

[2 INTRODUCTION: BACKGROUND INFORMATION AND SCIENTIFIC RATIONALE 9](#_Toc456962017)

[2.1 Background Information 9](#_Toc456962018)

[2.2 Rationale 9](#_Toc456962019)

[2.3 Potential Risks and Benefits 10](#_Toc456962020)

[2.3.1 Potential Risks 11](#_Toc456962021)

[2.3.2 Potential Benefits 11](#_Toc456962022)

[3 OBJECTIVES 14](#_Toc456962023)

[3.1 Study Objectives 14](#_Toc456962024)

[3.2 Study Outcome Measures 15](#_Toc456962025)

[3.2.1 Primary 15](#_Toc456962026)

[3.2.2 Secondary 15](#_Toc456962027)

[4 STUDY DESIGN 17](#_Toc456962028)

[5 STUDY ENROLLMENT AND WITHDRAWAL 18](#_Toc456962029)

[5.1 2. Subject Inclusion & Exclusion Criteria 18](#_Toc456962030)

[5.1.1 Randomization Procedures (if applicable) 19](#_Toc456962031)

[5.2 Premature Termination or Suspension of Study 19](#_Toc456962032)

[6 STUDY INTERVENTION 20](#_Toc456962033)

[6.1 Study Product Description 20](#_Toc456962034)

[6.1.1 Acquisition 20](#_Toc456962035)

[6.1.2 Formulation, Packaging, and Labeling 20](#_Toc456962036)

[6.1.3 Product Storage and Stability 20](#_Toc456962037)

[6.2 Dosage, Preparation and Administration of Study Product 20](#_Toc456962038)

[6.3 Assessment of Subject Compliance with Study Product Administration 20](#_Toc456962039)

[6.4 Concomitant Medications/Treatments 20](#_Toc456962040)

[7 STUDY SCHEDULE 21](#_Toc456962041)

[8 STUDY PROCEDURES /EVALUATIONS 22](#_Toc456962042)

[8.1 Laboratory Procedures/Evaluations 22](#_Toc456962043)

[8.1.1 Clinical Laboratory Evaluations 22](#_Toc456962044)

[9 ASSESSMENT OF SAFETY 23](#_Toc456962045)

[10 STATISTICAL CONSIDERATIONS 24](#_Toc456962046),24

[11 SOURCE DOCUMENTS AND ACCESS TO SOURCE DATA/DOCUMENTS 26](#_Toc456962047)

[12 ETHICS/PROTECTION OF HUMAN SUBJECTS 27](#_Toc456962049)

[12.1 Ethical Standard 27](#_Toc456962050)

[12.2 Institutional Review Board 27](#_Toc456962051)

[12.3 Informed Consent Process 27](#_Toc456962052)

[12.4 Exclusion of Women, Minorities, and Children (Special Populations)](#_Toc456962053) ...........................24

[12.5 Subject Confidentiality 27](#_Toc456962054)

[12.6 Future Use of Stored Specimens and Other Identifiable Data 28](#_Toc456962055)

[13 DATA HANDLING AND RECORD KEEPING 29](#_Toc456962056)

[13.1 Data Management Responsibilities 29](#_Toc456962057)

[13.2 Data Capture Methods 29](#_Toc456962058)

[13.3 Types of Data 29](#_Toc456962059)

[13.4 Schedule and Content of Reports 26](#_Toc456962060)

[13.5 Study Records Retention 29](#_Toc456962061)

[14 LITERATURE REFERENCES 31](#_Toc456962064)

15 APPENDICES LIST …………………………………………………………………………………………………………..……….33

LIST OF ABBREVIATIONS

| AE | Adverse Event/Adverse Experience |
| --- | --- |
| CONSORT | Consolidated Standards of Reporting Trials |
| CRF | Case Report Form |
| CPLE | *Carica papaya* leaf extract |
| GCP | Good Clinical Practice |
| ICF | Informed Consent Form |
| ICH | International Conference on Harmonization |
| IRB | Institutional Review Board |
| LFT | Liver Function Tests |
| AST | Aspartate Aminotransferase |
| ALT | Alanine Aminotransferase |
| MOP | Manual of Procedures |
| N | Number (typically refers to subjects) |
| PI | Principal Investigator |
| SAE | Serious Adverse Event/Serious Adverse Experience |
| SOP | Standard Operating Procedure |
| UP | Unanticipated Problem |
| WHO | World Health Organization |

PROTOCOL SUMMARY

| **Title:** | A prospective study to evaluate the safety and efficacy of *Carica papaya* leaf extract (oral CPLE (Caripill)) in the treatment of severe thrombocytopenia (≤30,000/µl) in dengue |
| --- | --- |
| **Précis:** | This will be a placebo-controlled, single center, randomized prospective study of CPLE (Caripill) (*Carica papaya* leaf extract) tabs in dengue patients with severe thrombocytopenia (platelets ≤ 30,000/ul). The study will enroll 100 eligible dengue cases into the two arms of the study – one arm receiving CPLE (Caripill) 1100mg *t.i.d* for 5 days and the other group receiving placebo. |
| **Objectives:** | Primary: To determine the impact of CPLE (Caripill) administration (1100mg *t.i.d* for 5 days on the following:   - Platelet count and hematocrit values in dengue patients with severe thrombocytopenia. - Reducing the number of blood product transfusions *esp.* platelet transfusion and - All-cause mortality rates. |
|  | Secondary: To evaluate the effect of CPLE (Caripill) administration (1100mg *t.i.d* for 5 days) on the following-   - Magnitude and kinetics of serum viremia (or NS1 antigenemia). - Magnitude and kinetics of cytokines IFNγ, IL6, TNFα and IL4. - The total length of hospital (ICU & ward) stay, |
| **Population:** | Study cohort of 100 adults, either gender (more than 18 yrs old, non-pregnant) and admitted to AIMS Hospital and diagnosed of dengue with moderate to severe thrombocytopenia. |
| **Number of Sites:** | Dept. of General Medicine and Dept. of Emergency Medicine, Amrita Institute of Medical Sciences and Research Center, Ponekkara, AIMS, Kochi-682041. |
| **Description of Intervention:** | Orally available CPLE (Caripill) Tabs (Micro Labs Ltd) at a dose of 1100 mg/tab three times a day for 5 days |
| **Study Duration:** | The estimated time (in months) from when the study opens to enrollment until completion of data analyses will be 12 months. |
| **Subject Participation Duration:** | Duration of hospital stay and the subsequent follow-up at 2 week post last dose of CPLE (Caripill). |
| **Estimated Time to Complete Enrollment:** | Immediately after screening positive and signing the informed consent form (ICF). |

**Schematic of Study Design**

For 50 cases: Obtain informed consent. Screen potential subjects by inclusion and exclusion criteria; obtain history and fill in the Clinical Research Form (CRF).

**Randomization**

**Treatment Group (N=50)**

**Control Group (N=50)**

**Day 0**

Clinical examination and detailed biochemical, hematological and virological laboratory investigations will be performed at baseline. Peripheral blood (5ml) will be collected; CRF will be completed for each enrolled subject. Administer CPLE (Caripill) or placebo *t.i.d* for 5 days.

Repeat study intervention (*if applicable*).

Follow-up assessments of study endpoints and safety. Appropriate recommended “standard of care” treatment modalities will be employed for each study subject. Routine hematological and biochemical laboratory assays will be done. Administer CPLE (Caripill) or placebo *t.i.d* for 4 more days.

**Day 1**

Follow-up assessments of study endpoints and safety. Appropriate recommended ‘standard of care” treatment modalities will be employed for each study subject. Routine hematological, biochemical and virological laboratory assays will be done. Administration of CPLE (Caripill) or placebo *t.i.d* will continue.

**Day 2**

Follow-up assessments of study endpoints and safety. Appropriate recommended ‘standard of care” treatment modalities will be employed for each study subject. Routine hematological and biochemical laboratory assays will be done. Administration of CPLE (Caripill) or placebo *t.i.d* will continue.

**Day 3**

Follow-up assessments of study endpoints and safety. Appropriate recommended ‘standard of care” treatment modalities will be employed for each study subject. Routine hematological and biochemical laboratory assays will be done. Administration of CPLE (Caripill) or placebo *t.i.d* will continue

**Day 4**

Final assessments of study endpoints and safety. Appropriate recommended ‘standard of care” treatment modalities will be employed for each study subject. Routine hematological and biochemical laboratory assays will be done. Administration of final dose of CPLE (Caripill) or placebo *t.i.d* continue

**Day 5**

Follow up assessments will occur at 2 week interval after the final dose of CPLE (Caripill) or placebo tab including the routine clinical, biochemical, hematological assays.

1. KEY ROLES AND CONTACT INFORMATION

| **Principal Investigator:** | **Dr. Vidya P.Menon.** MD DNB, FACP ; Associate Professor, Dept of General Medicine  Amrita Institute of Medical Sciences and Research Center,  AIMS Ponekkara, Kochi -682041. |
| --- | --- |
| **Co-PIs** | **Dr. C. Ravindranath Kamath**. BAMS, MBBS, FPM, Dept of Integrated Medicine  Amrita Institute of Medical Sciences and Research Center,  AIMS Ponekkara, Kochi-682041.  **Dr. Gireesh Kumar K.P.** MD (internal Medicine); Clinical Professor, Head of Dept of Emergency Medicine, Internal Medicine.  Amrita Institute of Medical Sciences and Research Center,  AIMS Ponekkara, Kochi-682041. |
| **Clinical Site Investigators:** | **Dr.Dipu T.S.** MD; Clinical Asst. Professor, Dept of General Medicine  Amrita Institute of Medical Sciences and Research Center,  AIMS Ponekkara, Kochi -682041  **Dr. Sabarish B** MD; Clinical Asst. Professor, Dept of Emergency Medicine  Amrita Institute of Medical Sciences and Research Center,  AIMS Ponekkara, Kochi -682041.  **Dr. Sreekrishnan T.P.** MD; Clinical Asst. Professor, Dept of Emergency Medicine  Amrita Institute of Medical Sciences and Research Center,  AIMS Ponekkara, Kochi -682041.  **Dr. Merlin Moni MD**; Clinical Asst. Professor, Dept of General Medicine  Amrita Institute of Medical Sciences and Research Center,  AIMS Ponekkara, Kochi -682041.  **Dr.Binny P.P.** MD; Clinical Asst. Professor, Dept of General Medicine  Amrita Institute of Medical Sciences and Research Center,  AIMS Ponekkara, Kochi -682041 |
| **Laboratories:** | **Dr. Veena P. Menon** Ph.D; Professor School of Pharmacy and Dept. of Clinical Virology.  Amrita Institute of Medical Sciences and Research Center, AIMS Ponekkara, Kochi -682041.  **Athira P.P.** M. Pharm .; Research Associate, Clinical Virology Laboratory, Amrita Institute of Medical Sciences and Research Center, AIMS Ponekkara, Kochi -682041. |
| **Project and Data Management** | **Rekha M.J.** M.B.A ; Project Co-ordinator, Dept of Medical Administration,  Amrita Institute of Medical Sciences and Research Center,  AIMS Ponekkara, Kochi -682041.  **Fabia E.T.** M.Sc.; Data Management & Research Analysis, Dept of Allied Health Sciences,  Amrita Institute of Medical Sciences and Research Center,  AIMS Ponekkara, Kochi -682041.  **Preetha P.** R.N.; Nurse Co-ordinator, Dept of Medical Administration,  Amrita Institute of Medical Sciences and Research Center,  AIMS Ponekkara, Kochi -682041. |

1. INTRODUCTION: BACKGROUND INFORMATION AND SCIENTIFIC RATIONALE
   1. Background Information

Dengue is a mosquito borne flavivirus infection that occurs in the tropical and sub-tropical regions of the world. World Health Organization (WHO) lists dengue among one of the seventeen prioritized, neglected tropical diseases that affects more than a billion populations. According to its most recent epidemiological data, dengue has a yearly incidence of about 50-100 million cases, with 40% of the global population considered at risk for dengue^1^. As the vector habitat expands, dengue outbreaks have become a common occurrence in Asia, especially India. Recent data from WHO reports the case fatality rate of dengue at 2.5% and the estimated morbidity and mortality due to dengue infection as approximately 1,300 disability-adjusted life years (DALYs) per million^2^. These figures clearly highlight the severe impact dengue infection can have on the public healthcare costs and resources. In India, dengue is endemic or hyperendemic in most parts and the healthcare and economic cost of regular and isolated outbreaks can be very high as estimated in the recent study by *Shepard et al*^3^.

Dengue is known to be heterogeneous in its clinical presentation and the unpredictable nature of its severity and outcomes often proves to be a challenge to the clinician. While a majority of dengue infections resolve rapidly after presenting as mild asymptomatic febrile illness, a small percentage of dengue infected cases progress to severe disease characterized by excessive bleeding and plasma leakage that can eventually lead to shock (DSS) and death. Management of dengue is largely based on providing timely, appropriate and supportive treatment. In order to facilitate early diagnosis and timely management of severe dengue, the WHO in 2009 introduced a new set of criteria for its clinical classification based on its severity. Based on clinical severity criteria, dengue cases are now categorized as ‘dengue with’ or ‘dengue without’ warning signs and severe dengue^5^.

**Dengue and Thrombocytopenia**

Thrombocytopenia, marked by a platelet count less than 150,000/µl of blood, is one of the hallmark indicators of severity in dengue viral infections. Typically platelet counts decline steeply during the infection within the initial 4-6 days of onset of fever and revert back to normal levels in the consequent days as the fever subsides. During the phase of defervescence, plasma leakage due to increased vascular permeability could potentially exacerbate the condition, leading to life threatening dengue shock syndrome (DSS) in patients with hemorrhagic fever (DHF) ^6^. Previous studies have reported an association between depleted platelet levels and higher prevalence of clinical bleeding in patients with dengue viral infections^7^. Inadequate platelet levels that fail to suppress bleeding and initiate coagulation could also lead to the bleeding symptoms. One of the several mechanisms proposed to explain thrombocytopenia during the infection of DENV serotypes involves the inhibition of bone marrow progenitor cell proliferation and functions^8^. Decline of megakaryocyte progenitors in the bone marrow has been demonstrated during dengue viral infection in humanized mice, possibly indicating the suppression of platelet production leading to dengue induced thrombocytopenia^9^. Depletion of platelets during dengue viral infection can also be attributed to the activated immune response that destroys platelets by augmented apoptosis, lysis and antibodies^10^. The non-structural protein NS1 is expressed in infected cells, eliciting immune responses. DENV infections also lead to up-regulation of membrane bound Tissue Factor (TF) and activation of platelets. Coagulation and inflammatory pathways are initiated, leading to the release of pro-inflammatory cytokines like TNF-α and IL-6. Raised levels of TNF-α has been associated with hemorrhagic symptoms. Moreover, immune dysregulation can also lead to alterations in platelets functions. Plasma leakage from capillaries has also been associated to altered platelet functions^11^ Endothelial sequestration is another mechanism that leads to thrombocytopenia. Platelets increasingly attach to von-Willebrand factor (vWF) on vascular endothelial cells, resulting in small clogs in microcirculation and low levels of platelets in the peripheral blood^12^.

Management of thrombocytopenia in dengue using platelet transfusions have met with no significant improvements in relation to bleeding and was associated with increased length of hospitalization and side effects. According to the Dengue Fever management guidelines recommended by the Dept of Health, Govt. of Kerala, platelet transfusions are not advisable in the absence of bleeding. Platelet transfusions are to be initiated only when platelet counts are <10,000 without bleed or platelet count <50,000 with bleeding^13^. This clearly suggests that management of thrombocytopenia in severe dengue cases is a challenge. Prophylactic transfusions are also currently discouraged by W.H.O^14^.

***Carica papaya* leaf extract. (CPLE) and its role in dengue associated thrombocytopenia**

The anti- inflammatory, anti-tumor and anti-bacterial effects of *Carica papaya* leaf extracts have been demonstrated in several studies^15-17^. Traditionally it has been also used in many countries as an anti-pyretic. *Carica papaya* is rich in alkaloids, saponins, flavonoids, tannins and glycosides that mediate its various biological activities. *Caricapapaya* leaves are rich in cardiac glycosides, anthraquinones, carpaine, pseudocarpaine, and other phenolic compounds^18-19^. Previous studies on papaya leaves have shown that seed extract of *C.papaya* possesses pharmacological activities, including anthelminthic, antifertility, contraceptive etc. A hot-water extract of the leaves is taken orally as an anti-pyretic, and in the treatment of anemia and appetite stimulation^19, 20^. It is also effectively used as a natural remedy to treat dengue fever associated with thrombocytopenia^19^. *In vitro* hemolytic studies have shown that CPLE has erythrocyte stabilization potential and thereby prevent hemolysis^21^. *In-silico* energy binding studies using bioinformatic tools have indicated that flavanoid quercetin from *Carica papaya* could potentially inhibit the NS2B-NS3 protease which are required for viral assembly. This however, has yet to be confirmed experimentally^22^. A toxicity study conducted in Sprague-Dawley rats revealed that oral administration of *Carica papaya* leaves juice (CPLJ) was not safe ^23, 24^.

Other murine studies have also reported a platelet and RBC increasing effect with administration of leaf extracts of *Carica papaya*^25, 26^. A similar study by *Hettige et al* performed in Sri Lanka confirmed a significant increase in the platelet as well as the leukocyte count^27^. *Subenthiran S et al* showed that *Carica papaya* leaves juice (CPLJ) significantly accelerates the rate of increase in platelet count in patients with dengue fever and DHF^28^. They also report a significant increase in the expression levels of ALOX 12 and PTAFR genes in the interventional group compared to those who received just the standard treatment. Both these genes play a major role in hematopoiesis^29, 30^.

**Clinical trials of *Carica papaya* leaf extract (Oral CPLE (Caripill)) in Dengue**

More recently, a clinical study performed in Rajasthan, India on 400 dengue patients with thrombocytopenia (platelet <150,000/µl), assessing the effect of CPLE on the platelet counts concluded that administration of CPLE increased the platelet count without any side effects and/or complications otherwise associated with thrombocytopenia. Additionally, it was also noted that there was a decrease in the length of hospital stay as well as significant reduction in the need for platelet transfusions for dengue patients on CPLE tabs^31^. Another recent large, multicentric, double blind, placebo-controlled, randomized trial evaluating the efficacy and safety of CPLE (Oral Caripill) as an empirical therapy for thrombocytopenia with a platelet count of 30,000 – 100,000/µl in dengue patients showed excellent results. Patients in the treatment group had significant increase in the platelet count over the therapy duration. With very few adverse events recorded in the treatment group, Caripill was demonstrated to be safe and efficacious for the studied indication^32^. These evidences strongly suggest the benefits of *Carica papaya* leaf extract in the management of dengue related thrombocytopenia. However, the beneficial role of CPLE is still not been evaluated in the management of severe thrombocytopenia (< 30,000/µl) especially in cases where platelet transfusions are warranted. Also, while the literature is rich in *in-vitro*, preclinical and clinical effects of *Carica papaya* leaf extract on the platelet count, not much is evidences are available on the mechanistic basis of this effect. There is also not much evidence on the direct antiviral effect of *Carica papaya* leaf extract. In both the trial studies, observational data is not available on the viral load, kinetics or infection induced inflammation.

- 1. Rationale

There is no available antiviral therapeutic against dengue illness. Dengue fever management is mostly symptomatic and measures are usually target the management of severe symptoms including thrombocytopenia. With the hyperendemicity of dengue infection in India, and crunch due to supply and demand mismatch of platelet transfusions in our resource limited setting, research into availability of a cost effective alternative treatment modality of dengue associated severe thrombocytopenia is the need of the hour. In the light of growing body of in-vitro, preclinical and clinical studies evaluate the efficacy of *Carica papaya* leaf extract (CPLE) in improving the platelet numbers in peripheral circulation in mild and moderate dengue, we also aimed to estimate the efficacy and safety of CPLE (Caripill tabs) in increasing the platelet count in severe dengue, classified as per the WHO 2009 guidelines. CPLE as Caripill was chosen as the study drug for both scientific and pragmatic reasons. Recent evidences confirmed the safety and tolerability of CPLE when administered as Caripill tabs in dengue patients reduce incidence of side effect. CPLE both as generic and as Caripill tabs and will therefore be immediately available to patients if there is a positive result from the trial.

- 1. Potential Risks and Benefits
     1. Potential Risks
- No serious risks are anticipated for this product. Preclinical and pilot clinical studies have well established the safety of Caripill for human use. C. papaya leaf extract at a dose of up to 2000mg/kg body weight in rats was safe indicating that in human, a dose of up to 19 g of C. papaya leaf extract would be safe. Preclinical sub-acute oral toxicity studies on Sprague-Dawley rats showed that C. papaya leaf extract at a dose up to fourteen times the levels employed in practical use in traditional medicine could be considered safe as a medicinal agent^23-25^.
- The detailed product information (Product Monograph) is provided as Supplement (Appendix III).
- As mentioned in the product monograph, CPLE is contraindicated in males with prostate dysfunction (ie, BPH or prostate cancer); C. papaya should be avoided as it increases the iron absorption. Excess iron may increase oxidative stress, especially in the aging male. Iron overload may increase the risk of developing prostate cancer. Caripill should be used with caution in individuals with bleeding disorders or those taking blood thinning medications such as aspirin or warfarin. In view of this, to ensure patient safety, we will be excluding these patients.
- Patients will be monitored for any liver impairment. Routine biochemical, LFTs (Liver function tests) including serum albumin levels will be monitored on a daily basis. These parameters will be monitored throughout the study period i.e including the hospital stay and follow-up visits.

### 2.3.2 Potential Benefits

There are important benefits of the proposed Caripil treatment on the study population. A number of studies have demonstrated the effectiveness of Caripill in increasing the total as well as rate of platelet counts and hematocrit levels in dengue patients with mild to moderate. Give the high risk of hemorrhages and shock in severe dengue cases and its associated mortality, any potential benefit of improvement in thrombocytopenia will be highly beneficial.

1. OBJECTIVES
   1. Study Objectives

Thrombocytopenia is a well-known risk factor of fatality in severe dengue cases. A steady decline in platelet counts is characteristically associated with dengue illness severity. Thrombocytopenia coupled to severe vascular leakage and shock, usually around the time of defervescence can prove to be fatal. We hypothesize that administration of CPLE (Caripill) tab early in the course of dengue illness increase the platelet counts and reduce the likelihood of severe complications.

The clinical benefit of CPLE (Caripill) administration on the platelet counts in mild and moderate dengue have been shown before, this study will present an opportunity to specifically evaluate the effectiveness of CPLE (Caripill) administration in severe thrombocytopenic (≤ 30,000/µL) dengue cases. We will also investigate the effect of CPLE on the viremia (or NS1 antigenemia) as well as the infection induced immune response.

Primary Objectives:

1. To evaluate if CPLE (Caripill) administration (1100 mg three times a day for 5 days) in dengue patients with thrombocytopenia ≤30,000/µL is superior to placebo with respect to improvement in platelet counts and hematocrit values.
2. To evaluate if CPLE (Caripill) administration (1100mg three times a day for 5 days) in dengue patients with thrombocytopenia ≤30,000/µL is superior to placebo with respect in reducing the number of platelet transfusions needed in management.
3. To determine and compare the incidence of bleeding episodes during hospitalization between the treatment and placebo groups. Bleeding will be defined as mucosal bleeds such as subcutaneous bleeding, minor mucosal hemorrhages, sub-conjunctival hemorrhage, epistaxis, petechiae, or ecchymosis, positive tourniquet test.
4. To evaluate the final outcome of dengue illness (survival/mortality rates) between the treatment and placebo groups.

Secondary Objectives:

1. To correlate the changes in platelet counts to the clinical phase of dengue illness (febrile/critical (plasma leak)/ convalescent) between the treatment and placebo groups.
2. To compare and evaluate the viremia (or NS1 antigenemia) with peripheral platelet counts between the treatment and placebo groups.
3. To evaluate length of hospital (ICU & ward) stay in patients receiving CPLE (Caripill) for severe dengue.
4. To compare and evaluate the levels cytokines TNFα, IFNγ, IL4 and IL6, on day 0, and 3 of study period between the treatment and placebo groups.
   1. Study Outcome Measures
      1. Primary

The primary efficacy endpoints will be assessed by comparing the increase in the platelet counts and hematocrit levels during the hospital stay between the treatment and placebo groups. The peak platelet count and hematocrit will also be noted and compared between the treatment and the placebo group. Additionally, the frequency and type of blood product, more specifically platelet transfusions required to manage thrombocytopenia during the course of illness will be compared between the two study arms.

Safety and tolerability will also be evaluated by comparing the proportion of patients with any AE (adverse event), and with any SAEs, between the treatment arms. The incidence of bleeding episodes as assessed by clinical evaluation twice daily between the treatment and placebo groups will also be evaluated. Evidences of subcutaneous bleeding minor mucosal hemorrhages, sub-conjunctival hemorrhage, epistaxis, petechiae, or ecchymosis), positive tourniquet test, hypotension and internal organ bleeding, hematemesis, intracranial bleed and melena will be assessed.

- - 1. Secondary

Secondary outcomes that we plan to evaluate include parameters indicative of disease progression or worsening of illness. These will be compared between the two study arms.

1. To correlate the change in platelet counts and subsequent response with or without CPLE (Caripill) to the phase of dengue (febrile/critical (plasma leak)/ convalescent) We will study the correlation of the duration of moderate to severe thrombocytopenia with the phase of the dengue- febrile, critical-plasma leak, convalescence. Additionally we will compare both arms of the study to examine if the intervention in any of the three phases affected the duration/severity of thrombocytopenia.
2. The type of bleeding manifestation between the two arms of the study will be characterized. We will examine the evidence of (mucosal, GI, GU, vaginal, IC bleed etc.) bleeding with severity of thrombocytopenia and response to intervention by comparing both arms.
3. Date of admission and date of discharge or death will be noted in the CRFs and will be used to determine the length of hospital stay (ICU & Ward), in severe thrombocytopenic dengue patients receiving CPLE (Caripill) treatment compared to the placebo group.
4. Other additional exploratory outcomes, intended primarily to evaluate the infection induced inflammatory response will be performed. Viral antigenemia and host immune responses are important determinants of disease severity and clinical outcome. Therefore, changes in the kinetics and levels of cytokines in severe thrombocytopenic dengue patients receiving CPLE (Caripill) therapy will be compared to the placebo group.
5. STUDY DESIGN

This study is a randomized, placebo-controlled; double-blind trial investigating CPLE (Caripill) therapy in adult dengue patients with a thrombocytopenia of < 30,000/µL. Adults (≥ 18yrs of age, non-pregnant) and laboratory (+ NS1 ELISA/ + vRNA RTPCR)/+IgM or +IgG) confirmed diagnosis of dengue and with a peripheral platelet count of ≤ 30,000/µL will be enrolled into the study after signing an informed consent form.

The study patients will be followed for clinical and laboratory endpoints in hospital until study day 5 (or daily as out-patients from discharge to day 5) and reviewed at an outpatient visit at week 2.

The patients will be assigned to one of the two treatment arms:

1. Active drug: CPLE (Caripill) CPLE 1100mg *t.i.d* for 5 days.

2) Placebo: visually matched placebo *t.i.d* for 5 days.

Oral tablet of CPLE (Caripill) and placebo will be provided by Micro Labs Ltd with appropriate masking for a double blind study. On receipt the medications will be stored in accordance with the manufacturer’s recommendation in a secure area.

Enrolled patients will be randomly assigned in a 1:1 ratio to receive CPLE (Caripill) or placebo once daily for 5 days. Online randomization software will be used for patient assignment. Only the clinical trials pharmacist will have access to the randomization list.

Micro Labs Ltd will provide CPLE (Caripill) and visually matched placebo without charge. All other study staff will be blinded to treatment allocation until after completion of the study. Each enrolled patient was assigned the next available study code that corresponded to a prepackaged pack of study drug. Clinical data were captured on structured case report forms that were entered into a secure database.

1. STUDY ENROLLMENT AND WITHDRAWAL

Adult (≥ 18yrs) patients admitted to the Amrita Institute of Medical Sciences and Research Center and meeting all the inclusion and exclusion screening criteria of the study will be enrolled once signed informed consent is obtained. A total of 100 cases of dengue with a thrombocytopenia (peripheral platelet count of ≤30,000/µL) will be enrolled into the two arms of the study. The point of enrollment is defined as the time at which a patient has signed and dated the consent form.

**5.1 Subject Inclusion Criteria**

In order to be eligible to participate in this study, an individual must meet all of the following criteria:

1. Provide signed and dated informed consent form
2. Willing to comply with all study procedures and be available for the duration of the study
3. Male or female, aged ≥ 18 yrs of age. Clinically diagnosed of dengue and a peripheral platelet count of ≤30,000/µL.
4. A laboratory positive diagnosis of dengue confirmed by a positive NS1 or IgM or IgG ELISA test and/or detection of viral RNA by real time PCR assay.

**5.2** **Subject Exclusion Criteria**

Patients with one or more of the following criteria at enrolment will be excluded from the study:

- 1. Pregnancy and lactation.
  2. Patients who has been given steroids for thrombocytopenia and also those who have been transfused platelets before starting the drug
  3. Taking indigenous medicines.
  4. Alanine aminotransferase (ALT) >150 U/L

**5.3 Strategies for Recruitment and Retention**

All patients recruited will be in-patients and we therefore do not anticipate any withdrawals.

**5.4 Treatment Assignment Procedures**

- 1. 1. Randomization Procedures (if applicable)

We will randomly assign enrolled patients in a 1:1 ratio to receive CPLE (Caripill) (1100mg) or placebo once daily for 5 days. Block randomization of will be stratified by age and gender into block sizes of 4 or 6.

**5.4.2 Masking Procedures (if applicable)**

MicroLabs Ltd. will provided CPLE (Caripill) tabs and visually matched placebo without charge. Only the clinical trials pharmacist had access to the randomization list. All other study staff will be blinded to treatment allocation until after completion of the study and database locking.

**5.5 Subject Withdrawal**

Subjects may withdraw voluntarily from the study or the investigator may terminate a subject's participation if:-

- Any clinical adverse event (AE), laboratory abnormality, or other medical condition or situation occurs such that continued participation in the study would not be in the best interest of the subject.
- The subject meets an exclusion criterion (either newly developed or not previously recognized) that precludes further study participation.
  1. Premature Termination or Suspension of Study

This study may be suspended or prematurely terminated if there is sufficient reasonable cause. Written notification, documenting the reason for study suspension or termination, will be provided by the P.I.s If the study is prematurely terminated or suspended, the principal investigator will promptly inform the IRB and will provide the reason(s) for the termination or suspension.

Circumstances that may warrant termination include, but are not limited to:

- Determination of unexpected, significant, or unacceptable risk to subjects.
- Insufficient adherence to protocol requirements.
- Data that is not sufficiently complete and/or evaluable.
- Determination of futility.

1. STUDY INTERVENTION
   1. Study Product Description

Package Insert information on CPLE (Caripill) tabs

- - 1. Acquisition

CPLE (Caripill) and placebo tablets will be supplied by MicroLabs Ltd.

- - 1. Formulation, Packaging, and Labeling

Packaging and labeling of CPLE (Caripill)

- - 1. Product Storage and Stability

The CPLE (Caripill) tabs will be stored at room temperature, away from heat and direct light.

- 1. Dosage, Preparation and Administration of Study Product

CPLE (Caripill) and placebo tabs of 1100mg in strength will be administered orally three times a day for 5 days.

- 1. Assessment of Subject Compliance with Study Product Administration

Each study subject will be directly observed for patient compliance by pill counts by the study nurse co-ordinator.

- 1. Concomitant Medications/Treatments

The treatment plans including the details of the concomitant medications will be recorded in the case record form (CRFs – Appendix I).

1. STUDY SCHEDULE

Screening

All adult patients admitted to Gen Medicine with a history of fever (<72 hr) will be clinically and laboratory evaluated for dengue illness. Subsequent to obtaining and documenting an informed consent form, the study subject will be further screened for detailed laboratory diagnosis for dengue and reviewed clinically for satisfying all study inclusion and exclusion criteria. Details in Schedule of Events

Detailed history and clinical examination will be performed and data recorded in the study CRF. Baseline hematological and biochemical, virological parameters (as part of standard of care) will be sought and recorded in the CRF. Study subject will be administered the study medications on day 1 after confirmed diagnosis as per schedule. Clinical evaluations, laboratory investigations including viral load will be performed during hospital stay.

1. STUDY PROCEDURES /EVALUATIONS

**8.1 Clinical evaluation**

Patients will be followed by a study physician daily until discharge, and all signs and symptoms recorded in the case report form. Clinical management decisions will remain in the hands of the attending doctors, a structured treatment plan will be provided so as to document the dengue management details. In the event that shock or any other serious complication develops, the patient will be transferred to the appropriate ICU. Details of all adverse events will be recorded on specific forms, together with an assessment as to whether the event is likely to be related to any treatment received, and all serious events will be reported promptly to the IRB. Patients who are fit to be discharged on or after study day 5. All patients will be asked to attend a follow-up visit at week 2 and repeat and report the LFT (Liver function test) results via phone.

- 1. Laboratory Procedures/Evaluations
     1. Clinical Laboratory Evaluations

Hematocrit, platelet and CBC measurements will be carried out daily or more frequently if clinically indicated. These tests will be repeated at the follow-up visit. Renal and liver function tests, electrolytes and coagulation profiles, will be carried out at enrolment, 48 hours later, day 5 of illness and at the follow-up visit. If the ALT measured 48 hours after enrolment is greater than 250 U/l, the study drug will be discontinued. It should, however, be noted that hepatic dysfunction might be secondary to dengue infection and could be positively affected by CPLE (Caripill) therapy. Conventional serological and virological tests will be used to confirm dengue infection and identify the infecting serotype. For a small subset of patients (n=20) plasma samples collected at daily intervals until discharge (and daily until day 5 if discharged before day 5) will be assessed for NS1 antigenemia, and concentrations of various pro- and anti-inflammatory cytokines (TNF-α, IFN-γ, IL-6, IL-4).

1. ASSESSMENT OF SAFETY

All data on potential adverse events, if any, will be recorded in a questionnaire/checklist. Further safety assessment will be also be done by monitoring for vital signs, laboratory evaluations – CBC – LFTs –Renal Function Tests during the study period with a follow-up at 2 weeks after stopping the medications.

Adverse Event / Experience

Any untoward medical occurrence associated with the use of CPLE (Caripill) in severe thrombocytopenic dengue patients whether or not considered drug related will be recorded on a AE grading scale (– Grade 1 Mild; asymptomatic or mild symptoms; clinical or diagnostic observations only; no intervention indicated – Grade 2 Moderate; minimal, local or noninvasive intervention indicated; limiting age-appropriate instrumental ADL – Grade 3 Severe or medically significant but not immediately life threatening; hospitalization or prolongation of hospitalization indicated; disabling; limiting self care ADL – Grade 4 Life-threatening consequences; urgent intervention indicated. – Grade 5 Death related to AE). All Serious Adverse Events regardless of causality will be reported.

1. STATISTICAL CONSIDERATIONS

**10.1 Sample size**

This is an exploratory study focusing primarily on efficacy of CPLE (Caripill) and based on earlier efficacy studies in mild and moderate dengue, a target sample size of 50 patients in 2 cohorts was chosen for enrolment over one year of dengue season based on clinical judgement and feasibility considerations.

Specifically, this study will have 80% power to detect an increase in platelet count and hematocrit levels by 20% due to CPLE (Caripill) therapy.

**10.2:**  **Main Analyses**:

The analysis and reporting of the result will follow the CONSORT guidelines[www.consort-statement.org](http://www.consort-sta/). The analysis results of patient demographics and baseline outcomes variables (both primary and secondary) will be summarized using descriptive summary measures; expressed as mean (standard deviation) or median (minimum-maximum) for continuous variables and number (percent) for categorical variables. We will analyze patients in the treatment group to which they are allocated, according to the intention –to –treat principle. Any patient’s lost or follow-up will be censored at the time they are lost to follow-up. We anticipate very few patients will not complete their follow-up. We will compare the patients allocated to CPLE (Caripill) group with patients allocated to placebo. All analysis will be done using SPSS 20. The primary analysis population will include all patients randomized to placebo from cohort 1 and all patients (regardless of treatment assignment) from cohort 2 according to the intention-to-treat principle.

The proportion of patients with any adverse events, any serious adverse events, or specific adverse events will be summarized and compared between the treatment arms based on Fisher’s exact test.

**10.3: Sub-group Analyses:**

Pre-defined secondary endpoints will be compared between the two treatment arms based on linear regression for continuous endpoints, logistic regression for binary endpoints, and Cox regression for time-to-event endpoints. For laboratory markers, comparisons will be adjusted for the pre-dose value of the respective marker and the day of illness at enrolment; plasma cytokines and NS1-endpoints will additionally be adjusted for dengue illness phase.

A detailed statistical analysis plan will be finalized prior to unblinding the study data base.

**10.4: Interim Analyses:**

An interim efficacy analysis based on the primary outcome will occur when 30% of the patients have been followed. The Haybittle-Peto rule will be applied to evaluate the efficacy of between the two treatment groups i.e. interim analysis shows a probability of equal to, or less than 0.001 between the two groups.

1. SOURCE DOCUMENTS AND ACCESS TO SOURCE DATA/DOCUMENTS

The study personnel will complete CRFs (**Appendix I**) and complete all the hematological, biochemical and virological laboratory values via accessing the hospital’s electronic medical records. Source documentation supporting the trial information reported on the CRF will be filed at the P.I.’s office and made available for trial related monitoring, audits, IRB/IEC review and regulatory inspections when required. All study records/files will be retained as per regulatory requirements.

All study staff will undergo a training session prior to trial commencement to ensure consistency in trial procedures including data collection and reporting. An operating manual will detail all steps in the protocol. The study co-coordinator will review detailed monthly reports on screening, enrollment, patient follow-up, data transmission, thoroughness and completeness of data collection, and event rates, and will rapidly address identified issues. CRF data will be checked and validated at many levels.

1. ETHICS/PROTECTION OF HUMAN SUBJECTS
   1. Ethical Standard

The P.I. will ensure that this study is conducted in full conformity with the principles set forth in Indian Council of Medical Research (ICMR)*,* International Ethical Guidelines for Biomedical Research Involving Human Subjects (2002*)* and Institutional Ethics Committee at Amrita Institute of Medical Sciences and Research Center.

- 1. Institutional Review Board

The protocol, informed consent form(s), recruitment materials, and all subject materials will be submitted to the IRB for review and approval. Approval of both the protocol and the consent form must be obtained before any subject is enrolled. Any amendment to the protocol will require review and approval by the IRB before the changes are implemented in the study.

- 1. Informed Consent Process

Informed consent is a process that is initiated prior to the individual agreeing to participate in the study and continues throughout study participation. Extensive discussion of risks and possible benefits of study participation will be provided to subjects and their families. A consent form describing in detail the study procedures and risks will be given to the subject (Appendix IIA). Consent forms will be IRB-approved, and the subject is required to read and review the document or have the document read to him or her. The investigator or designee will explain the research study to the subject and answer any questions that may arise. The subject will sign the informed consent document prior to any study-related assessments or procedures. Subjects will be given the opportunity to discuss the study with their surrogates or think about it prior to agreeing to participate. They may withdraw consent at any time throughout the course of the study. A copy of the signed informed consent document will be given to subjects for their records. The rights and welfare of the subjects will be protected by emphasizing to them that the quality of their clinical care will not be adversely affected if they decline to participate in this study.

The consent process will be documented in the clinical or research record.

- 1. Subject Confidentiality

Subject confidentiality is strictly held in trust by the investigators, study staff, and the sponsor(s) and their agents. This confidentiality is extended to cover testing of biological samples and genetic tests in addition to any study information relating to subjects.

The study protocol, documentation, data and all other information generated will be held in strict confidence. No information concerning the study or the data will be released to any unauthorized third party without prior written approval of the sponsor.

- 1. Future Use of Stored Specimens and Other Identifiable Data

Any residual specimens or other identifiable data will be maintained after the study is complete. We will procure the appropriate signed consent from the study subject to agree to future use of his/her specimens, images, audio or video recordings. The blood and other specimens will be maintained in the clinical virology lab in the -80^0^C and -20^0^C freezers for 1 yr post study. , All the specimens will be coded, de-identified to protect the confidentiality of the study subject.

1. DATA HANDLING AND RECORD KEEPING

The P.I. will be responsible for ensuring the accuracy, completeness, legibility, and timeliness of the data reported. All source documents will be completed in a neat, legible manner to ensure accurate interpretation of data. The study team investigators will maintain adequate case histories of study subjects, including accurate case report forms (CRFs), and source documentation.

- 1. Data Management Responsibilities

Data collection and accurate documentation are the responsibility of the study staff under the supervision of the investigator. All source documents and laboratory reports must be reviewed by the study team and data entry staff, which will ensure that they are accurate and complete. Unanticipated problems and adverse events must be reviewed by the investigator or designee.

- 1. Data Capture Methods

The primary data will be captured onto a Case Report Form (CRF) by the clinical trial site investigators. The data will be abstracted into an electronic format and used to generate a Microsoft Excel clinical study database. The database will be password protection and data will be double checked on a weekly basis by the study co-ordinators.

- 1. Types of Data

Complete clinical and outcome measures of all cause mortality and length of hospital stay will be captured. Laboratory data *esp.* of hematological, biochemical and virological will be captured into the CRF. In addition, safety data *esp.* incidence of adverse events and SAE will be recorded.

- 1. Study Records Retention

Study records will be maintained for at least five years from the date of study initiation. These documents should be retained for a longer period, however, if required.

1. PUBLICATION/DATA SHARING POLICY

The P.I will be responsible for developing publication procedures and resolving authorship issues. The study team along with the sponsors will be allowed for primary presentation and/or primary publication of the results. No other publication is allowed before the primary publication. Any subsequent presentation or publication by a study participant (including for sub studies) must be approved by the P.I. and the sponsor and make reference to the study and the primary publication. The final decision to publish articles and their content will be made by the P.I after prior notice to the Sponsor, allowing their review and comments on all manuscripts, at least thirty days in advance of submission.

1. LITERATURE REFERENCES
2. Accelerating work to overcome the global impact of neglected tropical diseases: a roadmap for implementation. Geneva, World Health Organization, 2012 (WHO/HTM/NTD/2012.1).
3. WHO. Dengue and dengue hemorrhagic fever. Factsheet N0117, revised February 2015. Geneva, World Health Organization.
4. Bhatt S., Gething P.W., Brady O.J. *et al*. The global distribution and burden of dengue. *Nature* 2013; 496, 504-7.
5. Shepard D.S., Halasa Y.A.,Tyagi B.K., Adhish S.V., Nandan D., *et al*. Economic and disease burden of dengue illness in India. *Am. J. Trop. Med. Hyg.* 2014 Dec; 91(6):1235-42.
6. WHO/TDR. Dengue guidelines for diagnosis, treatment, prevention and control. New edition. Geneva, World Health Organization, 2009.
7. Makroo R, Raina V, Kumar P, Kanth R. Role of platelet transfusion in the management of dengue patients in a tertiary care hospital. *Asian J Transfus Sci.* 2007; 1(1):4. doi:10.4103/0973-6247.28065.
8. Murgue B, Cassar O, Guigon M, Chungue E. Dengue virus inhibits human hematopoietic progenitor growth in vitro. *J Infect Dis.* 1997; 175(6):1497-1501. *http://www.ncbi.nlm.nih.gov/pubmed/9180193.*
9. Sridharan A, Chen Q, Tang KF, Ooi EE, Hibberd ML, Chen J. Inhibition of megakaryocyte development in the bone marrow underlies dengue virus-induced thrombocytopenia in humanized mice. *J Virol.* 2013; 87(21):11648-11658. doi:10.1128/JVI.01156-13.
10. Weaver SC, Vasilakis N. Molecular evolution of dengue viruses: contributions of phylogenetics to understanding the history and epidemiology of the preeminent arboviral disease. *Infect Genet Evol.* 2009; 9(4):523-540. doi:10.1016/j.meegid.2009.02.003.
11. Azeredo EL, Zagne SM, Santiago MA, et al. Characterization of lymphocyte response and cytokine patterns in patients with dengue fever. *Immunobiology.* 2001; 204(4):494-507. doi:10.1078/0171-2985-00058.
12. Michels M, Alisjahbana B, De Groot PG, et al. Platelet function alterations in dengue are associated with plasma leakage. *Thromb Haemost.* 2014; 112 (2):352-362. doi:10.1160/TH14-01-0056.
13. Eapen C., Elwyn E., Goel A., John T.J. Hypothesis of mechanism of thrombocytopenia in severe dengue, providing clues to better therapy to save lives. *Curr Sci.* 2015; 108 (2):168-169.
14. Dengue Fever Management Guidelines, Ministry of Health and Family Welfare, Government of Kerala. 2016. http://dhs.kerala.gov.in/docs/pdf/cd030613b.pdf
15. Handbook for Clinical Management of Dengue. WHO, 2012. http://whqlibdoc.who.int/publications/2009.
16. Otsuki,N., Dang, N.H., Kumagai, E.,Kondo,A. et al. Aqueous extract of *Carica papaya* leaves exhibits anti-tumor activity and immunomodulatory effects. *Journal of Ethnopharmacology*. 2010; (127):760767.
17. Romasi, E.F., Karina,J., Parhusip, A.J.N. Antibacterial activity of papaya leaf extracts against pathogenic bacteria. *Makara Teknologi*, 2011. 15(2):173-77.
18. Owoyele, B.N., Adebukola, O.M., Fumilayo, A.A. et al. Antiinflammatory activities of ethanolic extract of *Carica papaya* leaves. Inflammopharmacology. 2008 ; (16):168-173.
19. Zunjar, V.,Mammed, D., Trivedi, B.M. et al. Pharmacognostic , physico-chemical, and phytochemical studies on *Carica papaya* Linn. Leaves. *Pharmacognosy Journal*. 2011(3):5-8.
20. Kardono, L.B.S., Artanti, N., Dewiyanti, I.D. et al. *Carica papaya L*., Selected Indonesian Medicinal Plants and Monograph and Description. Vol 1. Grasindo, Jakarta, pp.167-182.
21. Sathasivam, K., Ramanadham, S.,Mansor, S.M. et al. Thrombocyte counts in mice after the administration of papaya leaf suspension. *The Middle European Journal of Medicine*. 2009; (121):19-22.
22. Priyanga R., Pathmasiri R., et al. In vitro erythrocyte membrane stabilization properties of *Carica* *papaya* L. leaf extracts. *Pharmacognosy Res*. 2012; 4(4):196202.
23. Senthilvel P, Lavanya P, Kumar KM, et al. Flavonoid from *Carica papaya* inhibits NS2B-NS3 protease and prevents dengue 2 viral assembly. *Bioinformation*. 2013; 9 (18):889-895. doi:10.6026/97320630009889.
24. S. Z. Halim S, N. R. Abdullah N, A. Afzan A, et al. Acute toxicity study of *Carica papaya* leaf extract in Sprague Dawley rats; Journal of Medicinal Plants Research. 2011; Vol 5:pp1867-72.
25. Halim S.Z. et al. Repeated dose 28 days oral toxicity study of *Carica papaya* L. leaf extract in Sprague Dawley rats. Molecules. 2012; 17:4326-42. doi: 10.3390/molecules17044326; www.mdpi.com/journal/molecules.
26. Dharmarathna SLCA, Wickramasinghe S, Waduge RN, Rajapakse RPVJ, Kularatne SAM. Does *Carica papaya* leaf-extract increase the platelet count? An experimental study in a murine model. *Asian Pac J Trop Biomed.* 2013;3(9):720-724. doi:10.1016/S2221-1691(13)60145-8.
27. Yunita F. et al. The effect of Carica papaya L. leaves extract capsules on platelet count and hematocrit level in dengue fever patient. *Int.J.Med.Arom.Plants.* 2012; 4340, Vol 2(4): 573-78.
28. Hettige S. Salutary effects of carica papaya leaf extract in dengue fever patients – a pilot study ,Sri Lanka: Sri Lankan Family Physician; 2008; 2008,29,17-19.
29. Subenthiran S, Choon T, Cheong K, Thayan R, Teck M, Muniandy P et al. *Carica papaya* Leaves Juice significantly accelerates the rate of increase in platelet count among patients with dengue fever and dengue haemorrhagic fever. Evidence-Based Complementary and Alternative Medicine. 2013;2013:1-7.
30. Kaur G., Jalagadugula,G., Mao, G. et al. RUNX1/core binding factor A2 regulates platelet 12-lipoxygenase gene (ALOX12): Studies in human RUNX1 haplodeficiency. Blood.2010; 115(15): 3128-35.
31. Sinhalagoda, l., Chandi, A. et al. Does *Carica papaya* leaf extract increase the platelet count? An experimental study in murine model. Asian.Pac.J.Trop.Biomed. 2013;3(9):720-24.
32. Gadhiwal A.K., Ankit, B.S., Chahar, C. et al. Effect of *Carica papaya* Leaf extract capsule on platelet count in patients of dengue fever with thrombocytopenia. Journal of the Association of Physicians of India. 2016:Vol 64:22.
33. Kasture P.N., Nagabhushan K.H., Kumar A. A multicenteric, double blind, placebo controlled, randomized, prospective study to evaluate the efficacy and safety of Carica papaya leaf extract, as empirical therapy for thrombocytopenia associated with dengue fever. Journal of the Association of Physicians of India. 2016:Vol 64:15.

APPENDICES

1. Appendix I: Case Report Form
2. Appendix II [A]: Informed Consent Form (English/Malayalam)
3. Appendix III: Product Monograph.
4. Appendix IV: Schedule of Events
5. Appendix V: Safety Assessment Forms
